# Exome sequencing in multiplex families with left-sided cardiac defects has high yield for disease gene discovery

**DOI:** 10.1371/journal.pgen.1010236

**Published:** 2022-06-23

**Authors:** David M. Gordon, David Cunningham, Gloria Zender, Patrick J. Lawrence, Jacqueline S. Penaloza, Hui Lin, Sara M. Fitzgerald-Butt, Katherine Myers, Tiffany Duong, Donald J. Corsmeier, Jeffrey B. Gaither, Harkness C. Kuck, Saranga Wijeratne, Blythe Moreland, Benjamin J. Kelly, Baylor-Johns Hopkins Center for Mendelian Genomics, Vidu Garg, Peter White, Kim L. McBride

**Affiliations:** 1 Computational Genomics Group, The Steve and Cindy Rasmussen Institute for Genomic Medicine, Nationwide Children’s Hospital, Columbus, Ohio, United States of America; 2 Center for Cardiovascular Research and The Heart Center, Nationwide Children’s Hospital, Columbus, Ohio, United States of America; 3 Department of Pediatrics, College of Medicine, The Ohio State University, Columbus, Ohio, United States of America; Broad Institute, UNITED STATES

## Abstract

Congenital heart disease (CHD) is a common group of birth defects with a strong genetic contribution to their etiology, but historically the diagnostic yield from exome studies of isolated CHD has been low. Pleiotropy, variable expressivity, and the difficulty of accurately phenotyping newborns contribute to this problem. We hypothesized that performing exome sequencing on selected individuals in families with multiple members affected by left-sided CHD, then filtering variants by population frequency, *in silico* predictive algorithms, and phenotypic annotations from publicly available databases would increase this yield and generate a list of candidate disease-causing variants that would show a high validation rate. In eight of the nineteen families in our study (42%), we established a well-known gene/phenotype link for a candidate variant or performed confirmation of a candidate variant’s effect on protein function, including variants in genes not previously described or firmly established as disease genes in the body of CHD literature: *BMP10*, *CASZ1*, *ROCK1* and *SMYD1*. Two plausible variants in different genes were found to segregate in the same family in two instances suggesting oligogenic inheritance. These results highlight the need for functional validation and demonstrate that in the era of next-generation sequencing, multiplex families with isolated CHD can still bring high yield to the discovery of novel disease genes.

## Author summary

Congenital heart disease is a common group of birth defects that are a leading cause of death in children under one year of age. There is strong evidence that genetics plays a role in causing congenital heart disease. While studies using individual cases have identified causative genes for those with a heart defect when accompanied by other birth defects or intellectual disabilities, for individuals who have only a heart defect without other problems, a genetic cause can be found in fewer than 10%. In this study, we enrolled families where there was more than one individual with a heart defect. This allowed us to take advantage of inheritance by searching for potential disease-causing genetic variants in common among all affected individuals in the family. Among 19 families studied, we were able to find a plausible disease-causing variant in eight of them and identified new genes that may cause or contribute to the presence of a heart defect. Two families had potential disease-causing variants in two different genes. We designed assays to test if the variants led to altered function of the protein coded by the gene, demonstrating a functional consequence that support the gene and variant as contributing to the heart defect. These findings show that studying families may be more effective than using individuals to find causes of heart defects. In addition, this family-based method suggests that changes in more than one gene may be required for a heart defect to occur.

## Introduction

Congenital heart disease (CHD) is one of the most common congenital anomalies, affecting over a million newborns each year worldwide. [1,2] Roughly one quarter of individuals with CHD have associated problems (other birth defects, neurodevelopmental disability) and are considered syndromic, while the remaining majority have CHD as an isolated condition. There is a strong genetic component to CHD with differential rates between sexes and ethnicities, and increased recurrence rates over the general population. [3–5] Well-known causes of syndromic CHD include chromosome anomalies (aneuploidies such as monosomy X, trisomies 13, 18, and 21), copy number variants (deletion 22q11, 1p36) and single gene disorders (*PTPN11*, *KTMD2*). [6] The causes of isolated CHD have been more difficult to identify, with fewer genes discovered using traditional genetic methods of linkage (*ACTC1*, *GATA4*, *MYH6*, *NKX2-5*, *NOTCH1*). [7–11] More recently, the number of single genes associated with CHD has expanded due to large scale efforts such as the Pediatric Cardiovascular Genetics Consortium (PCGC), utilizing exome sequencing (ES) of cases. [12]

Early reports from the PCGC identified *de novo* disease causing variants in single genes in approximately 10% of their initial cohort of child-parent trios. [13] Many genes were previously known to be associated with syndromes or were genes whose mechanisms have been implicated in causing multiple congenital anomalies and neurodevelopmental disorders. Later scrutiny noted that some of these individuals could be diagnosed with specific syndromes on close clinical evaluation. [14] More recent studies from the PCGC and UK10K project have noted much lower rates in their trios of isolated CHD, and associated genes have usually comprised a different group than those with CHD due to a syndrome. [15,16]

These data highlight several difficulties facing the field. First, pleiotropic conditions and syndromes frequently have variable expressivity and not all features are observable early in life. Phenotyping, particularly in the newborn, can thus be challenging even by skilled clinical geneticists. Second, identifying genes or common developmental pathways and mechanisms that may cause isolated CHD is difficult, with a significantly lower yield than syndromic CHD cases. Lastly, the genetic architecture for isolated CHD is likely different from CHD caused as part of a syndrome and teasing them apart is more challenging in the face of phenotyping errors.

We sought to overcome some of these challenges by returning to a family-based approach to CHD gene discovery. Investigating multiple affected individuals in the same family (multiplex family) allows increased confidence the phenotype is an isolated, not syndromic, CHD. Co-segregation of variants with disease also adds another criterion in support of causality. With each additional affected family member and higher degree of relationship between affected members, the more likely the CHD is due to a single inherited allele versus that of an oligogenic cause, an environmental factor, or a combination of factors. In addition, while hypothetically less frequent, oligogenic etiologies in multiplex families may be more easily observable because the number of co-segregating variants decreases as the number of affected members increases.

This study focused on multiplex families with left sided CHD, comprising the phenotypes of aortic valve stenosis (AVS), bicuspid aortic valve (BAV), coarctation of the aorta (COA), and hypoplastic left heart syndrome (HLHS). Our results demonstrate the utility of family-based studies in CHD gene discovery. We describe the involvement of three novel genes in CHD: *BMP10*, *ROCK1* and *SMYD1*, firmly establish *CASZ1* as a CHD gene, and provide evidence supporting oligogenic CHD etiology.

## Results

### Exome sequencing

A total of 19 families with 48 affected and 54 unaffected individuals were recruited (see **Table A in S1 Text**). ES was performed on all affected individuals for whom samples were available. Unaffected family members were recruited and had Sanger sequencing performed to assess segregation of candidate variants.

As sequencing technology advanced during the study, we analyzed each sample with the current best-practice sequencing platform and methodology. To avoid batch effect in cases where samples were processed using different exome capture kits, we used bedtools [17] to intersect BED files appropriate for each kit and filtered out variants not contained in mutually covered regions. The intersection of these capture kit targeted regions covered 18,321 genes. The mean coverage of target bases was 73.8x (SD = 23.4x), and 81.1% (SD = 11.8%) of target bases had at least 20x coverage. Around 12,000 protein-coding genes have at least 90% breadth at the 20x coverage threshold, as measured across RefSeq exonic regions padded with 5 bp. This increases to ~15,000 genes at the 10x coverage threshold. Across these exonic regions, ~92% of bases meet the 10x coverage threshold.

### Variant filtering and candidate gene prioritization

Our data pipeline and variant analysis process were used to filter variants from an average of 4,384 per family in the raw VCF data to an average of 69 after annotation. To pass filtering, variants were required to be present in each of the affected family members that were sequenced, have a maximum minor allele frequency (MAF) across populations in both gnomAD genome and exome sequencing cohorts ≤ 0.01, and have a Phred-scaled CADD score ≥ 20. Prioritization of variants passing filtering was performed using relevance of known phenotypes for their gene in human and mouse, and supported by *in silico* predictive algorithms. Candidate variants with a potential functionally relevant impact were identified in 17 of 19 families. Of those, seven families had a strong candidate variant in a gene with well-known CHD etiology, or support through functional validation of the variants impact on protein activity (**Table 1**). Each of these families is discussed here, as are two variants functionally assessed with negative results. See **Table B in S1 Text** for additional candidate variants. Phenotypes of all families and their members sequenced are shown in **Table 2**. Family pedigrees are provided in **Fig A in S1 Text** denoted by family identifier.

**Table 1:** Genes with rare, predicted damaging variants identified by exome sequencing in multiple affected members within 19 families with left sided defects.

Family	Phenotype	Gene: variant	MAF	CADD	CHD	Model
16 *	HLHS, BAV	***CASZ1***: NM_001079843.2(CASZ1):c.73C>T (p.Arg25Cys)	0.0007	32.0	[14,18,19] [20]	[21,22]
154 *	AVS / BAV (3), PVS	***ROCK1***: NM_005406.2(ROCK1):c.2083A>T (p.Lys695*)	0	40.0	[14,23]	[24]
		***MCTP2***: NM_018349.3(MCTP2):c.65A>C (p.Asn22Thr)	0.0005	32.7	[14,25]	[25]
207 *	BAV (2)	***ROBO4***: NM_019055.5(ROBO4):c.1087G>C (p.Val363Leu)	0.0002	22.5	[26]	[26]
238 *	BAV, BAV / VSD	***CTBP2***: NM_022802.2(CTBP2):c.2156G>A (p.Arg719His)	0.00003	27.8	[14]	[27]
346 *	AVS / BAV (2)	***SMYD1***: NM_198274.3(SMYD1):c.1321C>T (p.Arg441Trp)	0.014 **	33.0	[14]	[28]
		***BMP10***: NM_014482.1(BMP10):c.625C>T (p.Arg209Cys)	0.012 **	26.1	[14]	[29]
368 *	COA / BAV / VSD, COA	***MYH6***: NM_002471.3(MYH6):c.733_734delinsCC (p.Phe245Pro)	0		[14,30,31]	[31]
439 *	AVS / BAV (2)	***MATR3***: NM_018834.5(MATR3):c.629A>T (p.Glu210Val)	0.000009	23.0	[32]	[32]
469 *	HLHS, BAV	***NOTCH1***: NM_017617.4(NOTCH1):c.2995G>A (p.Val999Met)	0.0003	24.3	[8,14,33,34]	
528	COA, BAV	***HEY1***: NM_012258.3(HEY1):c.800C>A (p.Ser267Tyr)	0.0001	28.7		[35]

Families are listed with phenotype of individuals, candidate gene with variant location, variant minor allele frequency, bioinformatic damaging prediction, and supporting evidence with references that show association between the gene and congenital heart disease, as well as reports of a relevant heart phenotype in model organisms.

* Identified variant and gene considered likely causative.

** MAF > 0.01 due to gnomAD 3.0 update

**Table 2:** Sequenced subjects and phenotypes by family.

Subject ID	Relationship	CHD Phenotypes	Age of Diagnosis	Sex	Race (Self reported)
Family 16					
38	Proband	HLHS	1 Day	Male	White
39	Mother	BAV	35 Years	Female	White
Family 19					
54	Proband	BAV	3 Years	Male	White
55	Sibling	BAV	3 Years	Male	White
67	Sibling	BAV, COA	< 4 Years	Male	White
Family 58					
160	Proband	HLHS	1 Day	Male	Asian
162	Father	BAV	Unknown	Male	Asian
168	Sibling	ARSA	7 Years	Female	Asian
Family 72					
196	Proband	AVS, BAV	Unknown	Male	White
199	Sibling	BAV	Unknown	Male	White
Family 91					
284	Proband	BAV	18 years	Female	White
286	Father	BAV	36 years	Male	White
Family 118					
376	Proband	COA	6 Months	Female	White
377	Mother	BAV	36 Years	Female	White
Family 154					
517	Proband	AVS, BAV	2 Years	Male	White
515	Mother	AVS, BAV	< 8 Years	Female	White
514	Sibling, twin, identical	AVS, BAV	2 Years	Male	White
518	Sibling, half, maternal	Mild pulmonary valve abnormality without stenosis	12 Years	Female	White
Family 207					
683	Proband	BAV	< 9 years	Male	White
686	Sibling	BAV, Left ventricular noncompaction, Accessory mitral valve tissue	< 14 years	Male	White
Family 238					
796	Proband	BAV, VSD, Aortic Root Dilation	3 Years	Female	White
799	Sibling	BAV, VSD	4 Days	Female	White
Family 241					
806	Proband	HLHS	1 Day	Male	White
807	Mother	Unusually calcified mitral valve	21 Years	Female	White
Family 346					
1128	Proband	AVS, BAV	7 Days	Male	White
1131	Sibling	AVS	4 Years	Female	White
Family 368					
1197	Proband	BAV, COA, VSD	0 Days	Female	White
1698	Sibling	COA	4 Years	Male	White
Family 400					
1339	Proband	COA	15 Years	Male	White
1302	Father	COA	Unknown	Male	White
1870	Niece	HLHS	0 Days	Female	White
Family 439					
1430	Proband	AVS	12 Years	Male	White
1432	Father	BAV, COA	13 Years	Male	White
Family 469					
1529	Proband	HLHS	0 Days	Female	White
1531	Father	AVS, BAV	26 Years	Male	White
1608	Sibling	AVS	2 Days	Female	White
Family 481					
1567	Proband	BAV	Unknown	Female	White
1571	Sibling	BAV	Unknown	Male	White
1601	Sibling	AVS, BAV, Aortic aneurysm	Unknown	Male	White
Family 512					
1699	Proband	HLHS	Prenatal dx	Female	White
1701	Father	Pulmonary valve stenosis	Unknown	Male	White
1822	Sibling	DORV, Pulmonary valve atresia, TGA	Prenatal dx	Male	White
Family 528					
1747	Proband	BAV, COA	3 Weeks	Female	White
1749	Father	BAV	26 Years	Male	White
Family 549					
1823	Proband	AS, BAV	5 Years	Male	White
1826	Sibling	BAV	15 Years	Male	White
1828	Grandfather, maternal	AS, BAV	Unknown	Male	White

### CASZ1 p.Arg25Cys variant in familial AVS/BAV and HLHS

**Fig 1:**
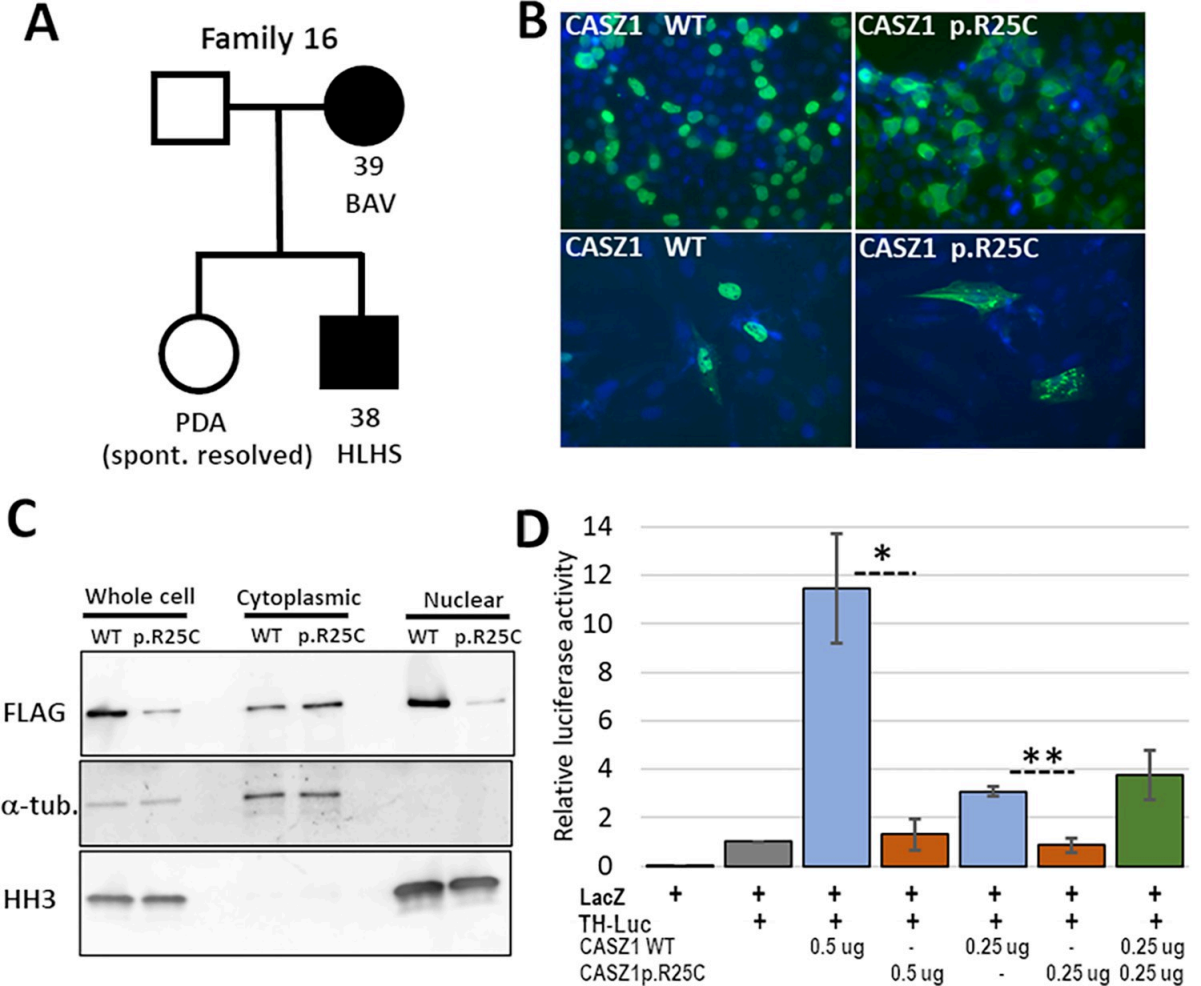
The CASZ1 p.R25C variant in family 16 causes mislocalization of the protein and reduced transcriptional transactivation. **A.** The pedigree of family 16, showing the two affected individuals, proband [38] and his affected mother [39], for whom ES was performed. **B.** IF staining for FLAG in HEK293T (top) and H9c2 (bottom) cells transfected with either FLAG-tagged CASZ1 WT or FLAG-tagged CASZ1 p.R25C. Note the predominately nuclear staining in both cell types with CASZ1 WT and the increased cytoplasmic staining in cells with CASZ1 p.R25C. **C.** Western blot analysis of unfractionated and fractionated protein extracts from HEK293T cells transfected with either FLAG-tagged CASZ1 WT or CASZ1 p.R25C, and probed with anti-FLAG antibody to detect tagged CASZ1, a-tubulin as a cytoplasmic marker, and histone H3 (HH3) as a nuclear marker. A majority of CASZ1 WT was present in the nuclear fraction. The CASZ1 p.R25C signal was higher in the cytoplasmic fraction, consistent with the IF staining results. **D.** A luciferase assay, using the tyrosine hydroxyylase (TH) promoter as a target on a luciferase expression construct in HEK293T cells, showed that cotransfection of CASZ1 WT increased luciferase reporter signal by over 10-fold, while CASZ1 p.R25C did not significantly increase luciferase signal above background. The effect of CASZ1 WT was dosage sensitive (compare 0.5 mg vs 0.25 mg), and cotransfection of CASZ1 WT and p.R25C was not significantly different from CASZ1 WT alone. The graphed data represent the combined results from three independent experiments in which each condition was performed in triplicate. (Error bars indicate SEM, * p = 0.013, ** p = 0.004).

Family 16 has three members with left sided defects, two of whom were available for sequencing (**Fig 1A and Fig A in S1 Text**). No individual had supravalvar aortic stenosis (SVAS) or cardiomyopathy. ES data were obtained for the proband and his mother and filtered for rare, damaging variants present in both individuals. Out of 100 variants that passed filtering, two met criteria as strong candidates in genes of interest *CASZ1* (NM_001079843.2:c.73C>T, p.Arg25Cys) and *ELN* (NM_000501.3:c.2132G>A, p.Gly711Asp).

CASZ1 (Castor Zinc Finger 1) is a zinc finger transcription factor required for normal heart development in the mouse [21,22]. Expression of mouse *Casz1* is detected throughout heart development from the formation of the cardiac crescent at E7.5 to adulthood, where it is most abundant in the myocardium. [21] Although expressed mainly in myocardium throughout development, *Casz1* is also detected in endocardium at E8.5. [21] Damaging variants in *CASZ1* were recently found to be associated with ventricular septal defect (VSD), dilated cardiomyopathy (DCM) and DCM with left ventricular noncompaction cardiomyopathy (LVNC). [18–20,36]

ELN (elastin) is a major extracellular matrix (ECM) protein in arteries. A recurrent deletion of 7q11.23 that results in loss of *ELN* causes Williams syndrome, [36] while heterozygous loss-of-function *ELN* variants are associated with a rare (1/20,000) familial SVAS. [37] BAV/AVS may also be found in roughly 10%, in conjunction with SVAS. *Eln* null mice die shortly after birth with obstructive arterial disease, [38] while *Eln*^*+/-*^ mice exhibit abnormal ECM remodeling in the aortic valve. [39] The ELN variant lies in a hydrophobic region of the elastin protein and non-conservatively replaces a glycine residue with aspartic acid. Loss of function *ELN* variants were recently identified in patients with left sided defects, conotruncal defects (CTD) or heterotaxy in the PCGC study, [14] however, *ELN* variants are not known to cause CHD in the absence of SVAS. Given the lack of SVAS in this family, the relatively high frequency (0.006) and presence of two homozygotes in gnomAD of the *ELN* variant, we thus focused on *CASZ1*.

The *CASZ1* p.Arg25Cys variant had sequencing coverage that was low at this position, under 10 total reads in both samples; however, Sanger sequencing confirmed the heterozygous genotype of both individuals. The variant was not found in any unaffected samples. The maximum population frequency of this variant is 0.0003, and it was predicted to be deleterious by CADD, SIFT, GERP++, and Polyphen2 Complex and Mendelian.

The *CASZ1* p.Arg25Cys variant was recently found in an embryonic rhabdomyosarcoma tumor. [40] Based on a previous study [41] that defined a CASZ1 nuclear localization signal (NLS) at amino acids 24–43, the variant was tested for its effect on CASZ1 subcellular localization in HEK293T cells and its ability to activate transcription of known target genes. CASZ1 p.Arg25Cys was found localized to the cytoplasm rather than the nucleus, and it failed to activate transcription of target genes. [40] We confirmed these results by IF staining, cell fractionation and a luciferase assay. We obtained the pCMV-Tag2A-CASZ1 expression construct that includes the full-length coding sequence for the human CASZ1b isoform with a FLAG tag fused to the N-terminus of the protein. [41] The *CASZ1* c.73C>T variant was introduced into the construct by site-directed mutagenesis, and the WT and variant plasmids were transiently transfected into HEK293T and H9c2 cells. The cells transfected with *CASZ1* WT generally showed strong staining in the nucleus, with less intense signal in the cytoplasm in both cell types (**Fig 1B**). By contrast, the CASZ1 p.Arg25Cys variant was detected at higher levels in the cytoplasm than in the nucleus.

To confirm these results, HEK293T cells that had been transfected with *CASZ1* WT or *CASZ1* c.73C>T were fractionated into nuclear and cytoplasmic extracts and analyzed by western blotting (**Fig 1C**). As expected, the CASZ1 WT signal was higher in the nuclear fraction, while the CASZ1 p.Arg25Cys variant protein was more abundant in the cytoplasmic fraction. We next tested whether the mislocalization of CASZ1 p.Arg25Cys to the cytoplasm affects its ability to activate transcription from a target promoter. A reporter construct, pGL-TH-Luc, that includes the human tyrosine hydroxylase (TH) promoter driving expression of luciferase was used as the target. [41,42] When co-transfected into HEK293T cells, CASZ1 WT increased transcription of the TH reporter ~10-fold above background, while CASZ1 p.Arg25Cys showed no significant stimulation of reporter expression above background (**Fig 1D**). Taken together, these results confirm that the *CASZ1* c.73C>T variant creates a hypomorphic allele, due to impaired transport of the protein into the nucleus and subsequent reduced activation of CASZ1 target genes.

### MCTP2 p.Asn22Thr and ROCK1 p.Lys695* stop gain in familial AVS/BAV

**Fig 2:**
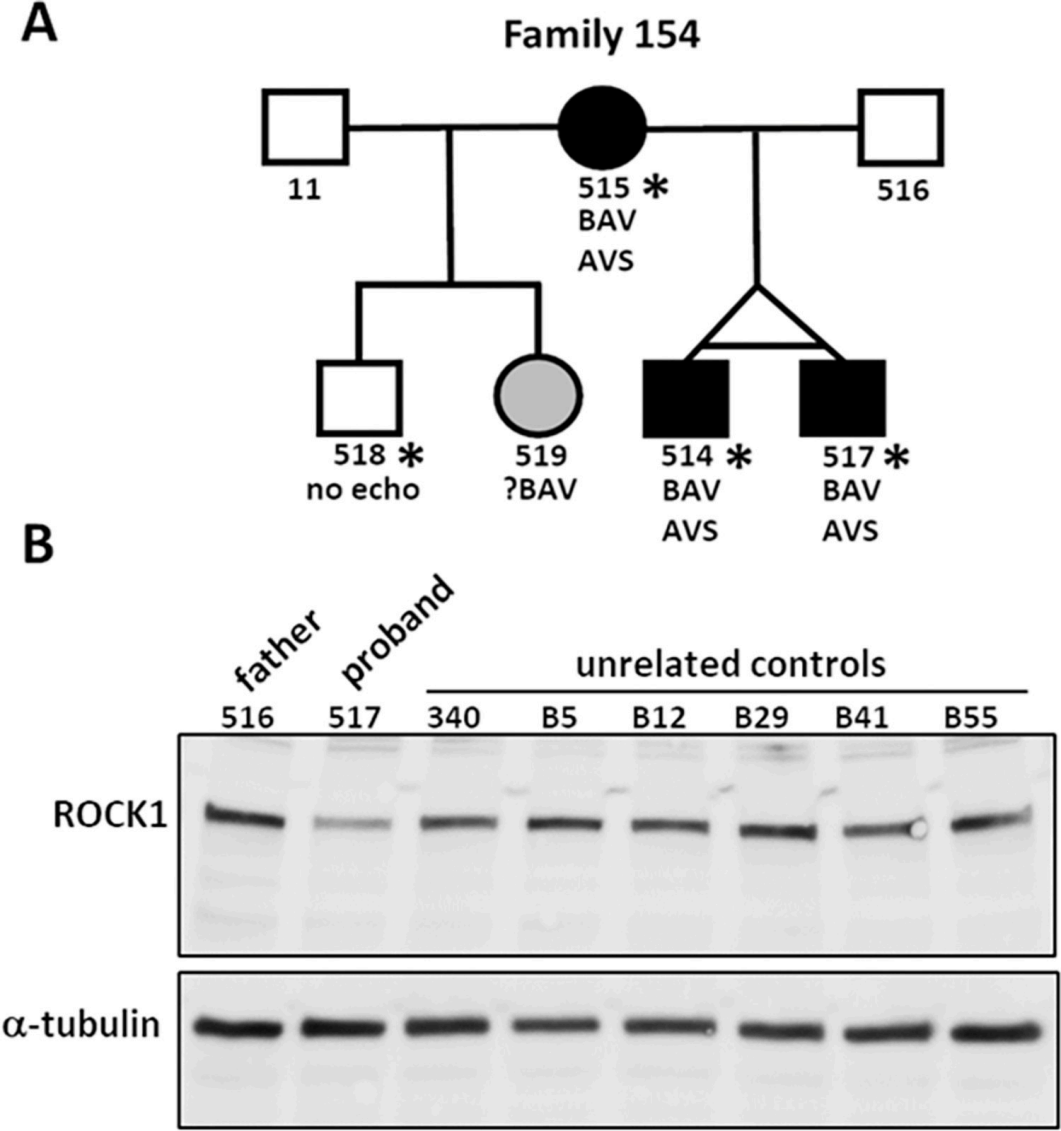
The heterozygous ROCK1 p.Lys695* stop gain variant in family 154 caused reduced expression of ROCK1 in the proband. **A.** Pedigree of family 154 showing the proband (517) and his affected identical twin brother (514) and their affected mother (515). ES was performed on individuals marked with an asterisk. **B.** A western blot of whole cell extracts from proband-derived lymphoblastoid cell lines (LCLs) and LCLs from seven unaffected individuals probed with anti-ROCK1 and anti-α-tubulin antibodies. Quantitation of ROCK1 signal relative to α-tubulin signal in each sample showed a ~50% reduction in ROCK1 in the proband relative to the mean value for the unaffected samples. The mean ROCK1/α-tubulin ratio for the unaffected samples was 0.53, SD±0.08, while the ratio in the proband sample was 0.23, greater than three standard deviations lower than the mean of the control samples.

Family 154 has three members with AVS/BAV and one with mild pulmonary valve anomaly without stenosis (**Fig 2A and Fig A in S1 Text**). Of 48 variants that passed filtering in family 154, two were considered candidates for contributing to the familial CHD: *ROCK1* NM_005406.2:c.2083A>T, p.Lys695*, with a CADD score of 40, and *MCTP2* NM_018349.3:c.65A>C, with a CADD score of 32.7 (**Table 1**). All four affected individuals were heterozygous for both variants. The *ROCK1* variant has not been reported in genomic databases, while the *MCTP2* p.Asn22Thr variant is present twice in gnomAD as is one occurrence of a p.Asn22Asp variant.

ROCK1 (Rho Associated Coiled-Coil Containing Protein Kinase 1) is a serine/threonine kinase that regulates cytoskeletal structure and dynamics through phosphorylation of multiple substrates, including myosin phosphatase (MYPT1) and myosin light chain (MYL). [42] Although constitutive knock-out of *Rock1* in mice is not associated with defects in heart development, [43] conditional expression of a dominant negative allele of *Rock1* in cardiac neural crest cells results in disorganization of the endocardial cushions and subsequent valve defects. [24] To investigate how the variant in family 154 might contribute to the CHD, we first considered whether the stop gain created by the *ROCK1* c.2083A>T variant affects the level of *ROCK1* mRNA through nonsense-mediated mRNA decay (NMD). RNA-Seq analysis of total RNA from LCLs derived from the proband (517), his unaffected father (516) and an unrelated control (340) showed that 17% (38/227) of reads for *ROCK1* in the proband included the c.2083A>T variant, while the samples from the father and control were 100% WT at this position (**Table C in S1 Text**). A western blot of whole-cell lysates from the proband’s LCLs and 7 unrelated WT LCLs probed with an antibody against the N-terminal domain of ROCK1 showed reduced expression of ROCK1 in the proband, with no evidence of a truncated protein expected at ~81 kD (**Fig 2B**). The average ROCK1/α-tubulin signal ratio of the control samples was 0.53 (SD = 0.08), while the ratio in the proband was 0.23, (>3 SD below the average of the control samples), indicating that the ROCK1 p.Lys695* variant results in a ~50% reduction in WT protein in heterozygous cells.

Although no truncated ROCK1 protein was detected in proband-derived LCLs by western blot, the presence of detectable (17%) variant *ROCK1* mRNA in these cells suggested that truncated protein could be present in quantities below the level of detection by western blot. We next examined what effect the truncated protein might have on the cytoskeletal structure. The ROCK1 kinase domain is located near the N-terminus of the protein (residues 71–399), followed by a coiled coil region, a Rho-binding domain (RBD) (residues 949–1014) and a plekstrin homology domain (PH) and cysteine-rich domain (CRD) near the C-terminus. ROCK1 kinase activity is activated either by binding of Rho GTPases to the RBD, or by caspase-3-mediated cleavage at Asp 1113 during apoptosis. Both these mechanisms are thought to activate the kinase by removing an autoinhibitory effect of the C-terminal PH and CRD domains. When ROCK1 is cleaved by caspase-3, its kinase activity becomes constitutively active, leading to an increase in myosin light chain phosphorylation and membrane blebbing. [43]

The *ROCK1* stop gain variant is predicted to leave the kinase domain intact, while eliminating the RBD and autoregulatory regions downstream. To determine whether ROCK1 p.Lys695* affects cell morphology, the c.2083A>T variant was introduced into the full-length WT mouse *Rock1* cDNA in the pCAG-6xMYC expression vector. MCF7 cells were transfected with the *Rock1* WT and c.2083A>T constructs and immunostained for the MYC tag after 13 h (**Fig BA in S1 Text**). The cells expressing the variant protein showed a highly irregular, condensed morphology, consistent with membrane blebbing, while the cells transfected with *Rock1* WT were morphologically similar to untransfected cells. Protein extracts from MCF7 cells transfected with the MYC-tagged expression constructs showed WT MYC-tagged Rock1 present at the expected higher molecular weight position (~170 kD) relative to endogenous ROCK1 (158 kD), and the MYC-tagged truncated Rock1 at ~100 kD (**Fig BB in S1 Text**). The presence of detectable truncated protein in cells transfected with the cDNA expression construct is likely because NMD is usually dependent on mRNA splicing. [44] Considering the dramatic dominant effect on cell morphology that was observed for transfected ROCK1 p.Lys695*, it is perhaps not surprising that it is not present at detectable levels in the proband LCLs.

MCTP2 (Multiple C2 Domain Containing Transmembrane Protein 2) is a calcium-binding protein associated with the ER, where it may participate in lipid biogenesis yet little is known about its function. [45] A previous report identified an inherited deletion on chromosome 15q26.2 that encompasses *MCTP2* in two half siblings with COA, and a *de novo* intragenic truncating duplication in *MCTP2* in a patient with HLHS and COA. [25] That study also found that knockdown of *Mctp2* in *Xenopus* embryos resulted in a lack of endocardial cushion formation, indicating an essential role for MCTP2 in heart development. More recently, heterozygous frameshift and stop gain variants in *MCTP2* were identified in two patients with left sided defects and one patient with TGA, [14] and also as a homozygote in one patient with AVS, PVS and neurodevelopmental disorder. [46]

At this time, we are not able to determine if one variant is causal or if both are required for the phenotype.

### SMYD1 p.Arg441Trp and BMP10 p.Arg209Cys variants in familial AVS/BAV

**Fig 3:**
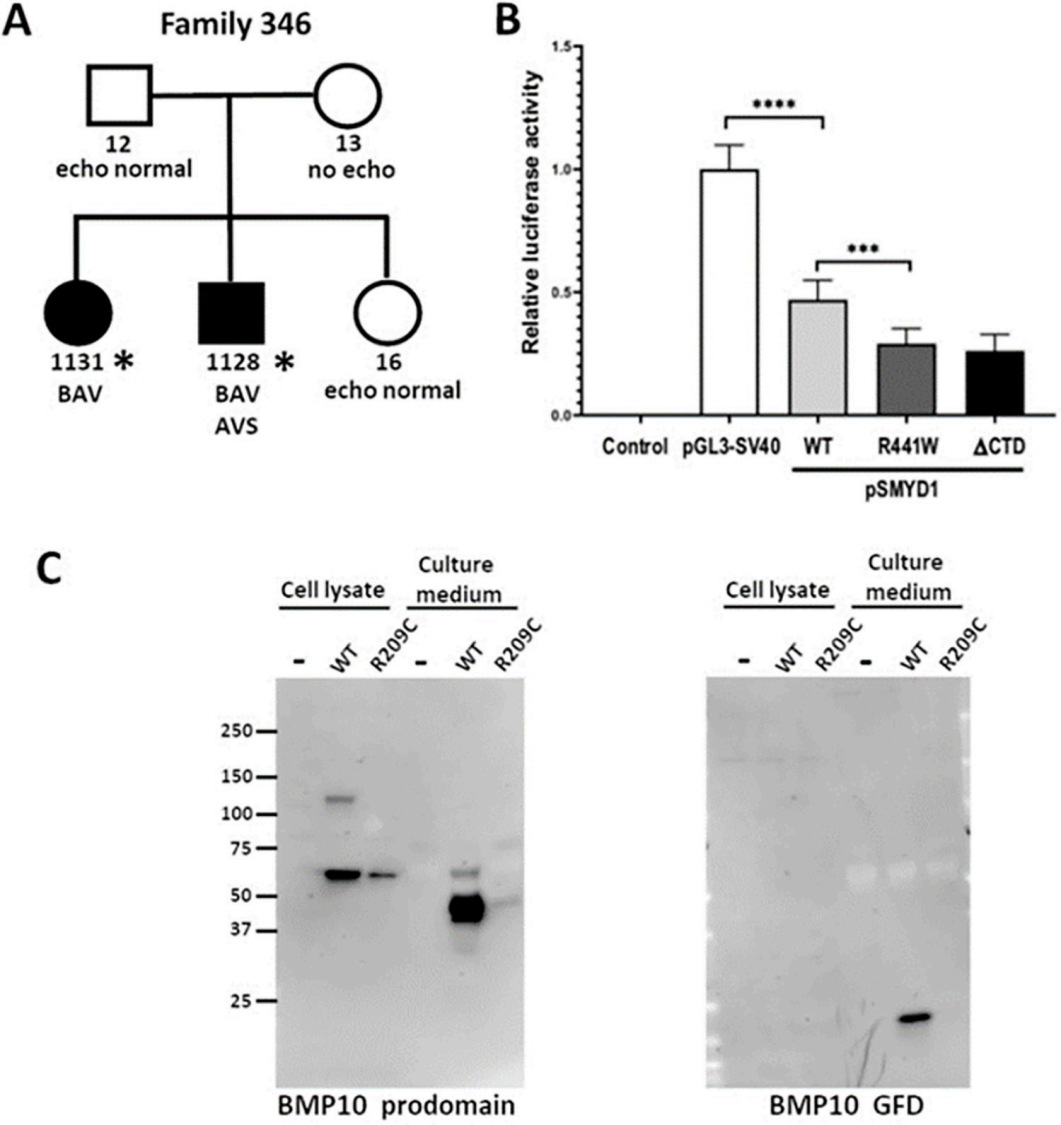
Rare variants in *SMYD1* and *BMP10* identified in family 346 are functionally damaging. **A.** ES was performed on two BAV patients from family 346 (asterisks). Both individuals were heterozygous for the SMYD1 p.R441W and BMP10 p.R209C variants. **B.** In a luciferase assay, using a reporter construct driven by the SV40 promoter (pGL3-SV40), cotransfection of a SMYD1 WT expression construct resulted in a 50% reduction in luciferase activity, demonstrating that SMYD1 represses transcription from the SV40 promoter. Transfection of the SMYD1 p.R441W variant protein caused significantly greater repression (~70%) of the reporter activity, to the same level as that of the SMYD1 DCTD variant that lacks the C-terminal autoinhibitory domain of SMYD1. These results indicate that SMYD1 p.R441W is a gain-of-function variant. The data shown represent the mean of values from 3 independent experiments in which each of the samples was assayed in triplicate (error bars indicate standard deviation, **** p<0.0001, *** p<0.001). **C**. Western blots of whole cell extracts or filtered culture medium from HEK293T cells stably transfected with BMP10 WT or BMP10 p.R209C expression constructs showed reduced levels of both the cellular (prodomain) and secreted form (growth factor domain) of the p.R209C variant protein.

Family 346 has two siblings with AVS and BAV and a distant maternal relative with BAV (**Fig 3A and Fig A in S1 Text**). Of the 89 variants in family 346 that passed analysis filters, two were considered strong candidates in genes of interest, *SMYD1* and *BMP10* (**Table 1**). The *SMYD1* variant (NM_198274.3:c.1321C>T, p.Arg441Trp) and *BMP10* variant (NM_014482.1:c.625C>T, p.Arg209Cys) were each heterozygous in both siblings, with the SMYD1 variant inherited from the father and BMP10 variant inherited from the mother. Both variants were predicted to be deleterious by CADD, SIFT, GERP++, and Polyphen2. The maximum population frequency at the time of variant filtering was 0.009 for *BMP10* p.Arg209Cys and 0.007 for *SMYD1* p.Arg441Trp. The probability of both variants occurring in the same individual is 0.000168 using population frequency in Table 1.

SMYD1 (SET And MYND Domain Containing 1) belongs to a family of lysine methyltransferases that regulate transcription through methylation of histone H3, thereby remodeling chromatin structure.[46] SMYD1 has no known DNA-binding domain, and it can act either as a transcriptional repressor or activator depending on its binding to other proteins, including histone deacetylases, skNAC and TRB3. [28,47,48] During mouse embryogenesis, *Smyd1* is expressed strongly in the developing heart and somites, and *Smyd1*^*-/-*^ embryos die by E10.5 with a single ventricle and increased extracellular matrix in the myocardium [28]. The protein includes a split SET domain that encodes the methyltransferase active site flanking a MYND domain that mediates protein-protein interactions, while a flexible 200 aa C-terminal domain (CTD) is thought to autoinhibit the methyltransferase activity. [28,49]

Previous studies found that SMYD1 associates with HDACs to repress transcription from the SV40 promoter *in vitro*. [28,48] We used a similar reporter assay to test whether the *SMYD1* p.Arg441Trp variant, lying within the CTD, affects the ability of SMYD1 to modulate promoter activity. SMYD1 expression constructs and the pGL3-SV40 luciferase reporter plasmid were co-transfected into HEK293T cells, and luciferase activity in cell lysates was measured 24h later (**Fig 3B**). SMYD1 WT reduced luciferase reporter activity by ~50%, while SMYD1 p.Arg441Trp showed a significantly greater repression of ~70%, a level equivalent to that produced by SMYD1 ΔCTD, which lacks the entire 200 aa CTD. Western blot analysis of whole-cell lysates from transfected HEK293T cells showed that the WT, p.Arg441Trp and ΔCTD forms of SMYD1 were present at equivalent levels, indicating that the variant does not destabilize the protein (**Fig C in S1 Text**). These results suggest that the p.Arg441Trp variant creates a gain-of-function allele through inactivation of the autoinhibitory function of the SMYD1 CTD.

BMP10 (Bone Morphogenetic Protein 10) is a member of the TGFβ superfamily of cytokines and is required for normal heart development in the mouse. [29,49] The protein is synthesized as a preproprotein that includes a signal sequence (aa 1–21), a prodomain (aa 22–316) and a growth factor domain (aa 317–424). During processing, the signal peptide is removed and the proprotein homodimerizes. The prodomain is cleaved from one of the subunits to yield a growth factor domain bound to a proprotein that is then secreted. [50]

To test whether the *BMP10* p.Arg209Cys variant is deleterious, the full-length *BMP10* WT coding sequence was cloned into a His-tagged expression vector, pcDNA3.1/V5-6xHis, and the *BMP10* c.625C>T variant was introduced into the construct by site-directed mutagenesis. Stably transfected HEK293T cell lines were generated for each construct and screened by qRT-PCR for clones that expressed either *BMP10* WT or *BMP10* c.625C>T at equivalent levels. Protein extracts were prepared from whole cells and the serum-free culture medium in which the cells were grown. Secreted BMP10 was partially purified and concentrated from the conditioned culture medium by affinity chromatography and ultrafiltration. When analyzed by western blots that were probed with an antibody to BMP10 propeptide, the level of variant p.Arg209Cys protein was lower than WT in whole cell extracts, as well as the culture medium (**Fig 3C**). This difference was more pronounced for the secreted BMP10 growth factor domain, where the WT protein showed robust signal while variant protein was nearly undetectable. We conclude that the *BMP10* p.Arg209Cys variant causes reduced levels of both the intracellular and secreted protein.

While we did not directly investigate the compound effects of variant pairs experimentally, *in silico* tools that predict digenic gene or variant interaction were run with inconclusive results, though this is a developing area of computational research (see **Supporting Materials and Methods in S1 Text**).

### Variants in genes previously described as causal for CHD

We identified likely causal variants in several genes previously implicated in CHD. These include *CTBP2* p.Arg719His in familial BAV/VSD, *MATR3* p.Glu210Val in familial AVS/BAV, *MYH6* p.Phe245Pro in familial COA, and *NOTCH1* p.Val999Met Variant in a family with BAV, AVS and HLHS, and *ROBO4* p.Val363Leu in familial BAV. Details are provided in the supplemental material (see **Supporting Results in S1 Text**).

### Negative functional studies

#### HEY1 p.Ser267Tyr variant in familial BAV with COA

Family 528 has a proband with COA and BAV and father with BAV (**Fig A in S1 Text**). Of the 91 variants that passed filtering in family 528, a *HEY1* variant (NM_012258.3:c.800C>A, p.Ser267Tyr) was considered the top candidate. The variant has a maximum population frequency of 0.00008 and a CADD score of 28.7.

*HEY1* (Hes Related Family BHLH Transcription Factor With YRPW Motif 1) belongs to a family of three bHLH transcription factors (HEY1, HEY2, HEYL) that mainly act as repressors and whose expression is induced by Notch1 signaling during embryonic development of several organs, including the heart. [51] *Hey1* knockout (KO) mice are viable and fertile with no heart defects detected, [35] while *Hey2* KO mice are viable with ASD, VSD, and AV valve malformations. [52] However, a *Hey1*/*Hey2* double KO genotype is embryonic lethal at E10.5, with the heart showing a single ventricle, hypoplastic trabeculae, failure of EMT in the AV cushion and vascular defects, [52] while *Hey1*/*HeyL* double KO mice die shortly after birth with VSD and dysplastic tricuspid and pulmonary valves. [35] Together, these results indicate that Hey proteins play partially redundant roles during cardiac development.

A previous study demonstrated that in a luciferase assay, transcription from the atrial natriuretic factor (ANF) promoter is stimulated by exogenous GATA4 and GATA6, and that this activity is repressed by co-expression of either Hey1, Hey2 or HeyL. [35] To determine whether the *HEY1*: p.Ser267Tyr variant affected the protein’s function in transcriptional regulation, plasmids expressing either *HEY1* WT or *HEY1* p.Ser267Tyr were constructed and co-transfected into HEK293T cells with an ANF-luc reporter, along with GATA4 and GATA6 expression constructs. No difference in luciferase activity was found between cells transfected with *HEY1* WT and *HEY1* p.Ser267Tyr. This functional data does not support HEY1 as causal for CHD in this family but cannot fully exclude it. No other variants in any other genes met filtering criteria. (**Fig D in S1 Text**).

## Discussion

We recruited and sequenced members of 19 multiplex families to investigate genetic etiologies of familial nonsyndromic CHD. Our study identified a likely disease-causing variant in eight families (42% of analyzed cases) and three genes with previously undescribed CHD associations, confirming our hypothesis that a family-based approach to variant analysis and gene discovery remains a high yield experimental method. Our method of ES for multiple affected family members with Sanger sequencing of candidate variants in the wider family offers a cost-effective template for familial studies while maintaining the analysis power of ES larger families. This family-based design also identified possible oligogenic inheritance, something that is exceedingly difficult to accomplish with case-control designs. Of the three novel genes identified, each presents significant opportunity for improving scientific knowledge of CHD pathogenesis.

There are two proposed hypotheses for the pathogenic mechanism of left sided defects, in particular for HLHS. The initial theory was that a perturbation in developing cardiomyocytes caused HLHS. However, an emerging hypothesis is HLHS and left sided defects are due to defects in endothelial/endocardial development. Several genes identified in our families are primarily sarcomeric. *MYH6*, *SMYD1*, and *CASZ1* are primarily involved in cardiomyocyte development, with limited expression of *SMYD1* and *CASZ1* in developing endothelial/endocardial cells. Human data show a rare missense variant in *MYH6* is a strong risk factor for COA in the Icelandic population, suggesting it is also important in endothelial cell development. We also identified variants in genes prominent in endothelial and endocardial development. *MATR3*, *NOTCH1* and *ROBO4* are expressed in endothelial cells of the valves of the heart and major arteries with important roles in endothelial cell integrity and in EMT, with reduced expression in cardiomyocytes. *BMP10* has been shown to be important in endothelial integrity, endothelial cell and vascular development, while also possessing a role in cardiac chamber development. *CTBP2* acts as a transcriptional repressor, acting in part by regulating epithelial to mesenchymal transition, but a direct role in endothelial cells has not been established. The role of *ROCK1* in the etiology of left sided defects is also not clear, although it is involved in cardiac neural crest cell function and endocardial cushion development. Similarly, the function of *MCTP2* is not well characterized, but does appear important in calcium handling in endothelial cells. These data suggest the two hypotheses are not competing but complementary.

Our identification of the *CASZ1* p.Arg25Cys variant in affected members of family 16 is the first instance in which this gene has been associated with HLHS and BAV. An increased prevalence of BAV among first-degree relatives of HLHS patients has been well documented, suggesting shared genetic etiologies for these disorders. [5,53–56] Previous reports found *CASZ1* damaging variants associated with VSD, DCM and LVNC, [18–20] consistent with studies of *Casz1* constitutive and conditional knockout mice that showed reduced proliferation of embryonic cardiomyocytes, resulting in VSD and abnormally thin ventricular walls. [21,36,41] Activation of the tyrosine hydroxylase (TH) promoter by CASZ1 was first described in neuronal cells, [57] and it has since been used to assess how coding variants in *CASZ1* affect its ability to activate transcription. [19,40,41] TH homozygous knock out mice die at mid-gestation with atrial defects and disorganized ventricular cardiomyocytes. [57] [58] Recently, it was demonstrated that CASZ1 promotes differentiation in myoblasts through epigenetic modification and changes in chromatin structure that facilitate the expression of muscle regulatory factors. [40]

*ROCK1* shows significant constraint for loss of function variants in population databases (pLI = 1, gnomAD o/e = 0.12), suggesting that the loss of a functional allele is damaging. The *ROCK1* p.Lys695* truncating variant identified in family 154 is predicted to leave the N-terminal kinase domain intact, while the absence of the autoinhibitory C-terminal RB and PH domains would render its enzymatic activity unregulated. The blebbing phenotype we observed when *ROCK1* p.Lys695* was transiently expressed in MCF7 cells is consistent with previous reports describing the effects of constitutively activated ROCK1. [49,59,60] We speculate that *ROCK1* p.Lys695* heterozygous cells are viable due to suppressed expression of the variant allele, most likely by NMD of the transcript. While the function of ROCK1 in the heart has been studied extensively in the context of adult disease, where it contributes to fibrosis and apoptosis, [42] its role in heart development is only beginning to be understood. *Rock1* constitutive knockout mice survive to birth, with the most prominent phenotypes being omphalocele and unfused eyelids. [43] Conditional disruption of Rock1 function specifically in cardiomyocytes using an inducible dominant negative allele, *ROCK1DN*, results in a thinner ventricular wall that appears during embryogenesis and persists after birth. [61] [24] Expressing the *ROCK1DN* allele specifically in neural crest cells (NCCs) by crossing to *Wnt1-cre* mice results in a variety of valve defects, including BAV (21). The Rock1DN-expressing NCCs show abnormal morphology, migration, and distribution in the developing outflow cushions. While that study did not include a constitutively active Rock1, it demonstrated the requirement for normal Rock1 signaling in valve development. [8,33,62–64] We speculate that the extensive migration of NCCs during normal valve development may be particularly sensitive to cytoskeletal abnormalities caused by disruption of ROCK1 signaling, and thus the presence of even a very low level of constitutively activated ROCK1 may be sufficient to produce valve defects. It is not known how the *ROCK1* and *MCTP2* variants may act in concert to cause left sided defects.

SMYD1 functions within signaling pathways known to be required for heart development. Its expression is regulated by *GATA6* and *MEF2C*, and it in turn targets the expression of the *HAND2* and *IRX4* transcription factors. [28,48,65,66] To our knowledge, *SMYD1* p.Arg441Trp is the first SMYD1 variant associated with CHD to be functionally validated as damaging. A predicted splicing variant in *SMYD1* was previously reported in a proband with heterotaxy, but its effect on SMYD1 expression is not known. [14] How the Arg441Trp variant affects the expression downstream targets of SMYD1 is not clear. *Smyd1* null mice show reduced expression of *Hand2* and *Irx4* in the developing heart at E9.0, [28] however, no *in vivo* model for specific loss of the SMYD1 CTD has been described.

*BMP10* is a member of the TGF-β superfamily, whose members play key roles in differentiation and organ morphogenesis. [66] While much of the superfamily is ubiquitously expressed, BMP10 is expressed exclusively in the heart. [49] Regulation of BMP10 expression during cardiac development has been shown to affect cardiac growth and chamber maturation. [29] Secretion of BMP10 from the cardiomyocytes and uptake by endocardial cells activates Nkx2-5, a protein required for normal aortic valve development. [67,68] The Arg209Cys variant lies in the TGF-β propeptide domain that is important to BMP10 bioactivity. [68] This variant was shown here to cause a reduction in intracellular BMP10, and a near abolition of the secreted form, suggesting a loss of BMP10 signaling as the cause of aortic valve disease in that family. SMYD1 and BMP10 are not known to interact.

While studies of non- left sided CHDs have shown good yields in the past, ES in left sided families has traditionally been less successful at identifying causative variants. [69] The use of multiplex families in our study and a growing body of variant annotation data may explain the higher yield. In families where we were unable to identify a causative variant, we hypothesize that this may be due to variants in intergenic sequences not covered by ES, lack of scientific literature providing sufficient evidence to warrant functional variant evaluation, or oligogenic etiologies beyond the scope of this study to investigate.

Part of the inability to identify causes of left sided defects is due to the likely complex inheritance pattern. Our previous genetic epidemiology studies demonstrated that single genes may be the cause in some families, but in most others the more probable scenario is oligogenic, with a predicted pattern of variants in two to six genes required for the phenotype to manifest. [5] A previous study of left sided defects has also shown digenic inheritances for HLHS in multiplex families (*SAP130* and *PCDHA13*), with confirmation in mouse models. [70] We identified more than one variant meeting our criteria for possible disease-causing candidate in several families. The strongest support for oligogenic causes of the CHD is present for our family with *BMP10* and *SMYD1* variants. Similarly, both *MCTP2* and *ROCK1* individually represent reasonable candidate genes, but if both are required for the expression of a CHD will require study. Pathogenic variants in *ELN* cause supravalvar aortic stenosis but are not known to cause AVS/BAV, thus it is uncertain if it is a phenotype modifier in the family with a *CASZ1* variant. Our study demonstrates the facility of multiplex family sequencing in beginning to uncover possible oligogenic causes of left sided defects.

We must mention several limitations in our study, alongside future directions to address those limitations. First, some of our families were small. While segregation even in affected parent-child families was helpful to narrow our search space, it is not as powerful as having additional, and more distant, affected relatives. In addition, some family members with reported cardiovascular issues could not be definitively treated as affected due to issues such as pedigrees potentially showing reduced penetrance or lack of echocardiogram data. Additional recruitment and clinical testing of extended family could add increased power in these cases. Second, the study was not designed to find variants in non-coding regions or that occur during post-zygotic development. Those families that do not have an obvious candidate are being subjected to genome sequencing, and when available, to sequencing of somatic tissues from relevant heart tissue samples. Third, our functional characterization provides evidence for plausible causality, but does not delve deep into mechanism. Furthermore, the functional studies were *in vitro* so the findings may not translate to the developing human heart. This is especially relevant to the findings with *HEY1*. In particular, our families with more than one candidate will require *in vivo* animal model experiments using double mutant crosses to establish the roles of these genes together.

In conclusion, the functional variant evaluation and existing body of genetic knowledge around *CASZ1*, *ROCK1*, *SMYD1*, and *BMP10* support their classification as likely disease-causal in the families in our study. Strong candidate variants discovered in *CTBP2*, *MATR3*, *MYH6*, *MCTP2*, *NOTCH1* and *ROBO4* reinforce previously published results of their involvement in CHD. Several families segregate more than one reasonable candidate variant, supporting previous data that left sided defects are likely oligogenic in nature. Sequencing multiple affected individuals in multiplex families by targeted exome or genome sequencing provides a high-yield, cost-effective method of variant analysis and gene discovery.

## Materials and methods

### Ethics statement

Individuals with CHD and their family members (both affected with CHD and unaffected) were recruited under a protocol approved by the Nationwide Children’s Hospital (NCH) Institutional Review Board (protocol number IRB09-00339). Written informed consent was obtained from subjects 18 years of age or older. Written consent was obtained from parents or legally authorized representative for subjects under age 18 years of age.

### Human subjects

Children and adults with CHD were approached at their NCH Heart Center visit or were referred to us by other institutions. Additional family members were recruited through the index case. Medical records were reviewed, and cardiac defects were confirmed by echocardiography, operative note, or procedure report. We searched for any additional health problems, including the presence of other birth defects, specific syndrome diagnosis, or neurodevelopmental disorders. Detailed clinical descriptions are presented in **Supporting Note: Case Reports in S1 Text**.

We defined the malformations as follows: AVS–congenital obstruction of the systemic outflow tract at the level of the valve, including trileaflet or bileaflet aortic valve (BAV); COA–a hemodynamically significant narrowing of the thoracic aorta, usually distal to the left subclavian artery (may or may not also have hypoplasia of the aortic arch); HLHS–mitral valve atresia or stenosis and aortic valve atresia or stenosis with hypoplasia of the left ventricle and aortic arch.

We included individuals if their left sided cardiac defect was isolated or co-occurred with a BAV or ventricular septal defect (VSD). We excluded those individuals who had a complex cardiac defect (e.g., presence of left sided defect and second major cardiovascular malformation), had a known chromosomal abnormality, or were diagnosed with a specific clinical genetic syndrome.

Blood samples were obtained for DNA and to establish Epstein-Barr virus-derived lymphoblastoid cell lines. Some individuals provided saliva samples (Oragene) for DNA in lieu of a blood sample. DNA was extracted using the Gentra PureGene kit.

### Exome library construction and sequencing

Exome libraries for samples sequenced at the Nationwide Children’s Hospital Biomedical Genomics Core were captured with the Agilent Clinical Research Exome Kit v1 and sequenced on an Illumina HiSeq 2500 (2 x 96 bp) or 4000 (2 x 150 bp). All other samples were sequenced at the Baylor-Hopkins Center for Mendelian Genomics (BHCMG), cohort ID BH23. Libraries in this group were captured with the Human Genome Sequencing Center (HGSC) designed Core capture reagent (52Mb, NimbleGen) and sequenced on an Illumina HiSeq 2000/2500 (2 x 100 bp) (see **Tables A and B in S1 Text**).

### ES data pipeline

Primary analysis was performed using Illumina’s Real-Time Analysis software to convert raw imaging data on the sequencer to base calls with associated quality scores. Illumina’s bcl2fastq Conversion Software was used to convert the resulting base call files to FASTQ format in preparation for secondary analysis. The Churchill pipeline [71] was used for secondary analysis, implementing alignment to the GRCh38 human reference genome and the GATK Best Practice Workflow for germline short variant discovery. [72] Scripts developed in house were run for tertiary variant analysis, which used SnpEff v4.3p [73] to determine both the location of each variant in reference to nearby genes and the variant’s effect on gene transcripts in the RefSeq database. Each variant was then annotated with population allele frequency, gene information, and predictive scores for impact on protein function. Plausibly disease-causing variants with low sequencing coverage in genes of interest were rescued for downstream analysis if manual evaluation at the alignment level indicated a high probability of a non-artifactual variant call.

### Variant analysis

With the goal of identifying a list of candidate variants for further study, variants were filtered by population allele frequency (MAF ≤ 0.01 in gnomAD 2.1.1) [74], predicted functional impact (Phred-scaled CADD scores [75] from the dbNSFP database [76] (v4.0) parsed into three tiers, I [CADD ≥ 30], II [CADD ≥ 20], and III [CADD < 20]), and expected inheritance model. The study began prior to the release of gnomAD 3.0; candidate variants that originally passed filtering but now exceed 0.01 MAF remain included but are noted as such. A higher MAF was chosen based on previous inheritance studies [5] and exome sequencing of CHD families indicate left sided defects that show reduced penetrance and excess of variants with MAF of up to 0.05. [77] Our collected families also show reduced penetrance.

Candidate genes were further assessed for biologic plausibility. We chose genes that: 1) have been previously reported as causing CHD; 2) that have a mouse model that has a phenotype of CHD; or 3) that have been shown in animal models to be important in cardiac development. Candidate variants were required to be present in all affected individuals within a family, and to be absent from unaffected samples except in cases where reduced penetrance was suspected. Variants matching autosomal dominant inheritance patterns were analyzed for all families, while autosomal recessive and compound heterozygous patterns were also considered for families 19, 346, and 368 (see **Table B in S1 Text**. In all families with variants reported as potentially causative were orthogonally confirmed in the index case and all available affected and unaffected family members using PCR amplification and Sanger sequencing.

### Functional studies

Strong candidate variants identified by ES in genes not previously associated with CHD, or in genes known to be associated with CHD but with differences in phenotype from our families, were studied further. Variants in genes considered to be known CHD disease genes that were considered likely causal by ACMG criteria (with minor modification to incorporate reduced penetrance) were not studied. To test the hypothesis that variants were disease-causing in families 16 (*CASZ1*), 154 (*ROCK1*), 346 (*BMP10* and *SMYD1*), and 528 (*HEY1*), we created cell-based assays to assess the variants’ functional effect. A general description of the assays is provided here, with a more detailed description of the assays and reagents provided in the Supporting Materials and Methods in S1 Text.

### Plasmids

The full-length coding sequence of each of the candidates were either purchased or constructed and cloned into plasmids (see Supporting Materials and Methods in S1 Text) and appropriate reporters purchased or constructed. Site directed mutagenesis was performed for sequence variants *CASZ1*, *ROCK1*, *SMYD1*, *BMP10*, and *HEY1* to introduce each into their respective expression constructs. Sanger sequencing of the entire coding sequence in all the modified plasmids was used to verify the presence of the desired variant and absence of any additional sequence changes. A more detailed description of the methods used to construct the expression plasmids is provided in the Supporting Materials and Methods in S1 Text.

### Cell culture

Unless otherwise noted, all cell lines were cultured in DMEM (Gibco, 1056910) supplemented with 10% fetal bovine serum (Gibco, 26140079), penicillin/streptomycin (HyClone, SV0010) and 2 mM L-glutamine (Gibco, 25030–91) at 37°C in 5% CO_2_. For cells transfected with Lipofectamine 2000, antibiotics were omitted from the culture medium.

### Western blotting

Whole-cell protein extracts were prepared, and concentrations measured. Protein extracts were resolved by SDS-PAGE on polyacrylamide gradient gels and transferred to a PVDF blotting membrane. After probing the blots with primary antibody, blots were incubated with AP-conjugated secondary antibodies, followed by fluorescent signal capture and quantification. Primary antibodies are listed in the Supporting Materials and Methods in S1 Text.

### Immunofluorescence microscopy

Immunofluorescent microscopy was used for analysis of subcellular localization, as described in the Supporting Materials and Methods in S1 Text.

### Cell fractionation

Subcellular localization was performed for the *CASZ1* and *ROCK1* variants. One aliquot of transfected cells was prepared for whole cell measurements and a separate aliquot used for nuclei pelleting to obtain cytosolic and nuclear fractions. Western blots were probed as described in the Supporting Materials and Methods in S1 Text.

### Luciferase assays

Luciferase assays were performed using appropriate downstream reporter assays for CASZ1 (pGL4.1-TH-Luc), SMYD1 (pGL3-promoter [SV40]), and HEY1 (pGL2-ANF, pDEST27-GATA4 and pDEST27-GATA6) along with pCMV-LacZ. The luciferase signal was normalized to the β-galactosidase signal in each sample to control for variation in transfection efficiency. A detailed description of the assays is provided in the Supporting Materials and Methods in S1 Text.

### RNA-Seq

Total RNA was isolated from cultured lymphoblastoid cell lines (LCL). RNA was quality checked, rRNA removed, and cDNA synthesized to create libraries with the NEBNext Ultra II Directional kit. Libraries were pooled and sequenced at 2 x 150 bp read lengths on the Illumina HiSeq 4000 platform, followed by alignment and analysis of single nucleotide position metrics using bam-readcount.

### Expression studies

Protein expression was performed for ROCK1 using transfected MCF7 cells by Western blots of whole cell lysates. Expression of both cellular and secreted BMP10 was performed on stably transfected HEK293T cells, selected using Geneticin, by Western blotting of cell lysates and culture media (see Supporting Materials and Methods in S1 Text).

## Supporting information

S1 Text Pedigree diagrams, sequencing information by subject, supporting methods and materials, and case report data. FIG A. PEDIGREE DIAGRAMS FOR SEQUENCED FAMILIES, LABELED BY FAMILY NUMBER. Family 16. CASZ1: C.73C>T (P.ARG25CYS). Family 19. AKAP13, CHD8, KCNJ2 and CNTRL variants.Family 58. AGR3, CLDN20, SHF and MYOM2 variants. Family 72. KRIT1: C.499C>T (P.ARG167CYS). Family 91. XBP1 and AKAP13 variants. Family 118. MYH7B: C.1502T>A (P.PHE501TYR). Family 154. MCTP2 and ROCK1 variants. Family 207. MYOCD, ROBO4 and WHSC1 variants. Family 238. CTBP2: C.536G>A (P.ARG719HIS). Family 241. NRAP: C.4648C>T (P.ARG1550TRP). Family 346. SMYD1 and BMP10 variants. Family 368. MYH6: C.734T>C (P.PHE245SER). Family 439. MATR3: C.629A>T (P.GLU210VAL). Family 469. NOTCH1: C.2995G>A (P.VAL999MET). Family 481. C1ORF127 and VEZF1 variants. Family 512. DNAH5: C.1715T>G (P.LEU572TRP). Family 528. HEY1: C.800C>A (P.SER267TYR). Family 549. GJC1 and NUB1 variants. FIG B. ROCK1 P695* CAUSES BLEBBING IN MCF7 CELLS. **A.** MCF7 cells were transfected with either Rock1 p.K695*-MYC or Rock1 WT-MYC expression constructs and stained for the MYC tag after 13 h. Cells expressing the pK695* variant displayed a more compact, globular morphology than the WT-expressing cells, consistent with membrane blebbing associated with activated Rock1. **B.** No signal was detected at the expected size for Rock1 p.K695* in protein from LCLs derived from the proband heterozygous for the ROCK1 c2083A>T variant. In MCF7 cells transfected with MYC-tagged WT and pK695* Rock1 expression constructs, bands were detected at the expected sizes above and below endogenous Rock1. FIG C. FLAG-TAGGED SMYD1 WT, SMYD1 P.R441W, AND SMYD1 delta-CTD WERE DETECTED AT EQUIVALENT LEVELS BY WESTERN BLOT. Whole-cell extracts (20 μg/lane) from transfected and untransfected (-) HEK293T cells were analyzed in duplicate. The anti-FLAG antibody detected the expected 58 kD band in WT and p.R441W samples, and 35 kD band in the ΔCTD sample. The same blot was reprobed for histone H3 (HH3) as an endogenous reference. FIG D. HEY1 P.S276Y AND HEY1 WT SHOWED THE SAME LEVEL OF REPRESSION OF GATA4/6-INDUCED EXPRESSION FROM THE ANF PROMOTER IN A LUCIFERASE REPORTER ASSAY. HEK293T cells were co-transfected with a reporter plasmid that includes the luciferase gene under the control of the ANF promoter and pCMV-LacZ, as a control for transfection efficiency. Co-transfection of constructs that express GATA4 and GATA6 increased luciferase activity by greater than 10-fold. Co-transfection of a HEY1 WT expression construct completely abrogated the effect of GATA4/6 on luciferase activity. Co-transfection of a HEY1 p.S276Y expression construct resulted in repression of luciferase activity that was not significantly different from HEY1 WT. Graphed values represent luciferase activity relative to LacZ activity, normalized to the ANF-Luc control samples. Each bar represents the combined results from three independent experiments with triplicate samples in each experiment. Error bars represent the standard deviation. * P < 0.00001, ** P = 0.246. TABLE A. SEQUENCING INFORMATION BY SUBJECT. TABLE B. SUMMARY TABLE OF RARE DAMAGING. Genes with rare, damaging variants identified by ES in multiple affected members within 19 families with LVOT defects. References that show association between the gene and congenital heart disease (CHD), as well as reports of a relevant heart phenotype in model organisms are listed on the right. Numbers of individuals with variants in cases and controls in the PCGC dataset are noted in the two far right columns, as numbers of recessive, loss of function and de novo variants separated by “/” (cases n = 2871, controls n = 1789). TABLE C. RNA-SEQ ANALYSIS IN FAMILY 154. RNA-Seq analysis for the presence of the *ROCK1* c.2083T>A variant in RNA from LCLs derived from the proband (517) and his unaffected father (516) from family 154, and an unrelated control (5698). SUPPORTING RESULTS. SUPPORTING MATERIALS AND METHODS. SUPPORTING NOTE: CASE REPORTS.
(DOCX)
