# Supplementary material for: Exome sequencing in multiplex families with left-sided cardiac defects has high yield for disease gene discovery
Source: PLoS Genet. 2022 Jun 23;18(6):e1010236. doi: 10.1371/journal.pgen.1010236 (PMC9258875; doi:10.1371/journal.pgen.1010236)
Supplement: S1 Text — FIG A. PEDIGREE DIAGRAMS FOR SEQUENCED FAMILIES, LABELED BY FAMILY NUMBER. Family 16. CASZ1: C.73C>T (P.ARG25CYS). Family 19. AKAP13, CHD8, KCNJ2 and CNTRL variants.Family 58. AGR3, CLDN20, SHF and MYOM2 variants. Family 72. KRIT1: C.499C>T (P.ARG167CYS). Family 91. XBP1 and AKAP13 variants. Family 118. MYH7B: C.1502T>A (P.PHE501TYR). Family 154. MCTP2 and ROCK1 variants. Family 207. MYOCD, ROBO4 and WHSC1 variants. Family 238. CTBP2: C.536G>A (P.ARG719HIS). Family 241. NRAP: C.4648C>T (P.ARG1550TRP). Family 346. SMYD1 and BMP10 variants. Family 368. MYH6: C.734T>C (P.PHE245SER). Family 439. MATR3: C.629A>T (P.GLU210VAL). Family 469. NOTCH1: C.2995G>A (P.VAL999MET). Family 481. C1ORF127 and VEZF1 variants. Family 512. DNAH5: C.1715T>G (P.LEU572TRP). Family 528. HEY1: C.800C>A (P.SER267TYR). Family 549. GJC1 and NUB1 variants. FIG B. ROCK1 P695* CAUSES BLEBBING IN MCF7 CELLS. A. MCF7 cells were transfected with either Rock1 p.K695*-MYC or Rock1 WT-MYC expression constructs and stained for the MYC tag after 13 h. Cells expressing the pK695* variant displayed a more compact, globular morphology than the WT-expressing cells, consistent with membrane blebbing associated with activated Rock1. B. No signal was detected at the expected size for Rock1 p.K695* in protein from LCLs derived from the proband heterozygous for the ROCK1 c2083A>T variant. In MCF7 cells transfected with MYC-tagged WT and pK695* Rock1 expression constructs, bands were detected at the expected sizes above and below endogenous Rock1. FIG C. FLAG-TAGGED SMYD1 WT, SMYD1 P.R441W, AND SMYD1 delta-CTD WERE DETECTED AT EQUIVALENT LEVELS BY WESTERN BLOT. Whole-cell extracts (20 μg/lane) from transfected and untransfected (-) HEK293T cells were analyzed in duplicate. The anti-FLAG antibody detected the expected 58 kD band in WT and p.R441W samples, and 35 kD band in the ΔCTD sample. The same blot was reprobed for histone H3 (HH3) as an endogenous reference. FIG D. HEY1 P.S276Y AND HEY1 WT SHOWED THE SAME LEVEL [file pgen.1010236.s001.docx]

**--- Supporting Information ---**

**Exome sequencing in multiplex families with left-sided cardiac defects has high yield for disease gene discovery**

David M. Gordon^1¶^, David Cunningham^2¶^, Gloria Zender^2^, Patrick J. Lawrence^1,2^, Jacqueline S. Penaloza^1^, Hui Lin^2^, Sara M. Fitzgerald-Butt^2,3^, Katherine Myers^2^, Tiffany Duong^2^, Donald J. Corsmeier^1^, Jeffrey B. Gaither^1^, Harkness C. Kuck^1^, Saranga Wijeratne^1^, Blythe Moreland^1^, Benjamin J. Kelly^1^, Baylor-Johns Hopkins Center for Mendelian Genomics, Vidu Garg^2,3^, Peter White^1,3^, Kim L. McBride^2,3*^

^1^ Computational Genomics Group, The Steve and Cindy Rasmussen Institute for Genomic Medicine, Nationwide Children’s Hospital, Columbus, Ohio, 43205, USA

^2^ Center for Cardiovascular Research and The Heart Center, Nationwide Children’s Hospital, Columbus, Ohio, 43205, USA

^3^ Department of Pediatrics, College of Medicine, The Ohio State University, Columbus, Ohio, 43210, USA

^¶^ These authors contributed equally to this work

^*^ Correspondence: [kim.mcbride@nationwidechildrens.org](mailto:kim.mcbride@nationwidechildrens.org)

Short Title: **Disease gene discovery in families with left-sided cardiac defects**

**Table of Contents**

[Figure A. Pedigree diagrams for sequenced families, labeled by family number. 4](#_Toc104277073)

[Family 16.](#_Toc104277074) *[CASZ1](#_Toc104277074)*[: c.73C>T (p.Arg25Cys). 4](#_Toc104277074)

[Family 19. AKAP13, CHD8, KCNJ2 and CNTRL variants. 5](#_Toc104277075)

[Family 58. AGR3, CLDN20, SHF and MYOM2 variants. 6](#_Toc104277076)

[Family 72. *KRIT1*: c.499C>T (p.Arg167Cys). 7](#_Toc104277077)

[Family 91. *XBP1* and *AKAP13* variants. 8](#_Toc104277078)

[Family 118. *MYH7B*: c.1502T>A (p.Phe501Tyr). 9](#_Toc104277079)

[Family 154. *MCTP2* and *ROCK1* variants. 10](#_Toc104277080)

[Family 207. *MYOCD*, *ROBO4* and *WHSC1* variants. 11](#_Toc104277081)

[Family 238. *CTBP2*: c.536G>A (p.Arg719His). 12](#_Toc104277082)

[Family 241. *NRAP*: c.4648C>T (p.Arg1550Trp). 13](#_Toc104277083)

[Family 346. *SMYD1* and BMP10 variants. 14](#_Toc104277084)

[Family 368. *MYH6*: c.734T>C (p.Phe245Ser). 15](#_Toc104277085)

[Family 439. *MATR3*: c.629A>T (p.Glu210Val). 17](#_Toc104277086)

[Family 469. *NOTCH1*: c.2995G>A (p.Val999Met). 18](#_Toc104277087)

[Family 481. *C1orf127* and *VEZF1* variants. 19](#_Toc104277088)

[Family 512. *DNAH5*: c.1715T>G (p.Leu572Trp). 20](#_Toc104277089)

[Family 528. *HEY1*: c.800C>A (p.Ser267Tyr). 21](#_Toc104277090)

[Family 549. *GJC1* and *NUB1* variants. 22](#_Toc104277091)

[Figure B. Rock1 p695* causes blebbing in MCF7 cells. 23](#_Toc104277092)

[Figure C. FLAG-tagged SMYD1 WT, SMYD1 p.R441W, and SMYD1 ΔCTD were detected at equivalent levels by western blot. 24](#_Toc104277093)

[Figure D. HEY1 p.S276Y and HEY1 WT showed the same level of repression of GATA4/6-induced expression from the ANF promoter in a luciferase reporter assay. 25](#_Toc104277094)

[Table A. Sequencing information by subject. 26](#_Toc104277095)

[Table B. Summary table of rare damaging variants. 28](#_Toc104277096)

[Table C. RNA-Seq analysis in Family 154. 30](#_Toc104277097)

[Supporting Results 31](#_Toc104277098)

[ROBO4 p.Val363Leu Variant in Familial BAV 31](#_Toc104277099)

[CTBP2 p.Arg719His Variant in Familial BAV/VSD 31](#_Toc104277100)

[MYH6 p.Phe245Pro Variant in Familial COA 32](#_Toc104277101)

[MATR3 p.Glu210Val Variant in Familial AVS/BAV 32](#_Toc104277102)

[NOTCH1 p.Val999Met Variant in Familial AVS with HLHS and BAV Cases 33](#_Toc104277103)

[Supporting Materials and Methods 34](#_Toc104277104)

[Clinical Evaluation 34](#_Toc104277105)

[Plasmids 34](#_Toc104277106)

[Site-directed mutagenesis 35](#_Toc104277107)

[Western Blotting 35](#_Toc104277108)

[Antibodies 36](#_Toc104277109)

[Immunofluorescence microscopy 36](#_Toc104277110)

[Cell fractionation 36](#_Toc104277111)

[Luciferase assays 37](#_Toc104277112)

[RNA-Seq 38](#_Toc104277113)

[*Rock1* expression 38](#_Toc104277114)

[*BMP10* Stable Transfectants 39](#_Toc104277115)

[*BMP10* expression and secretion 39](#_Toc104277116)

[*In silico* assessment of digenic variant interaction 40](#_Toc104277117)

[Supporting Note: Case Reports 41](#_Toc104277118)

#
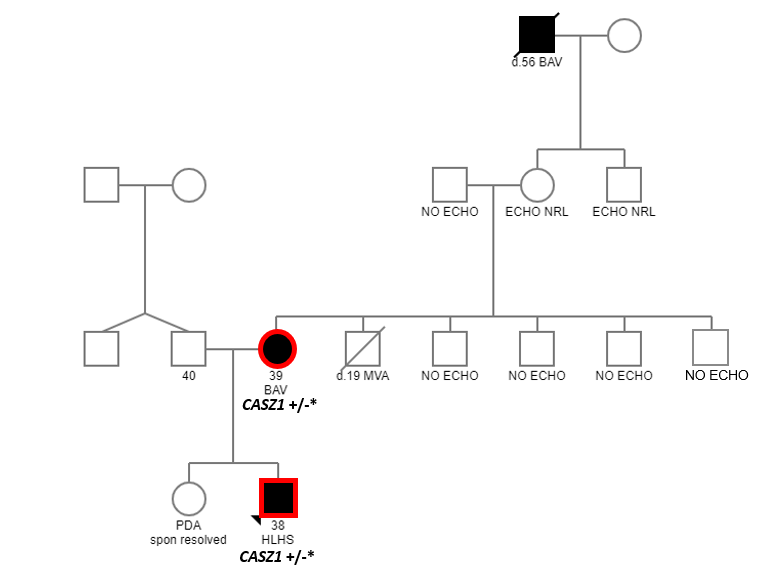
Figure A. Pedigree diagrams for sequenced families, labeled by family number.

Family 16. *CASZ1*: c.73C>T (p.Arg25Cys). Exome sequencing (ES) is denoted by red outline. Sanger validation is denoted as *.


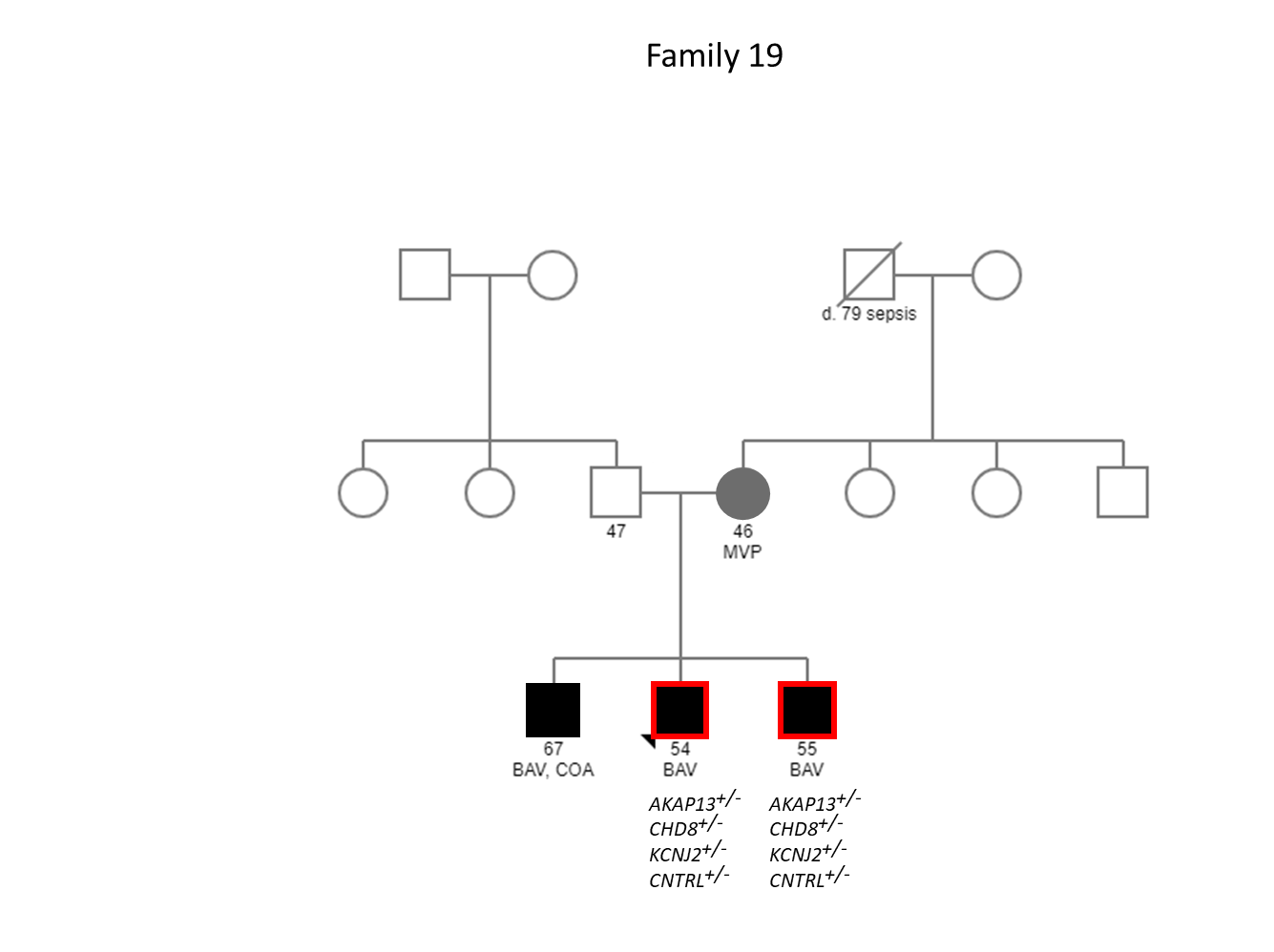


Family 19. AKAP13, CHD8, KCNJ2 and CNTRL variants. ES is denoted by red outline. *AKAP13*: c.4594G>A:p.Asp1532Asn; *CHD8*: c.7258C>T:p.Arg2420Cys; *KCNJ2*: c.863A>T:p.Asp288Val; *CNTRL*: c.38C>A:p.Ala13Glu


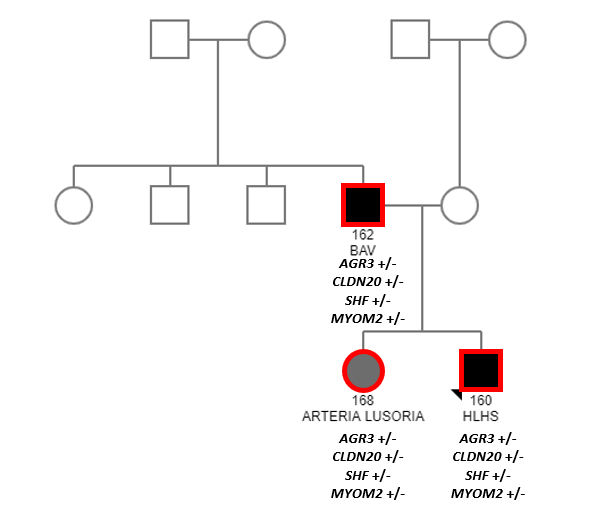


Family 58. AGR3, CLDN20, SHF and MYOM2 variants. ES is denoted by red outline. Sanger Validation was not performed. ***AGR3*: c.400G>T (p.Gly134*). *CLDN20*: c.316C>T (p.Arg106Cys). *SHF*: c.527C>T (p.Pro176Leu). *MYOM2*: c.1355C>T (p.Ser452Phe).**


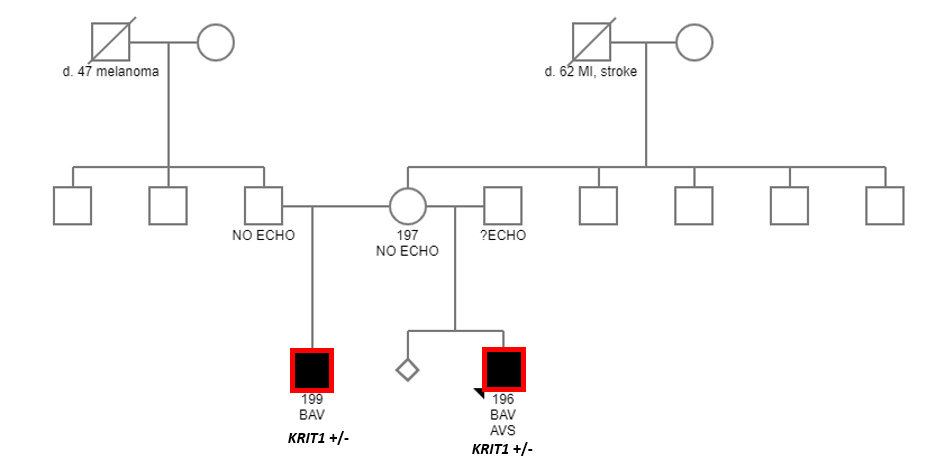


Family 72. *KRIT1*: c.499C>T (p.Arg167Cys). ES is denoted by red outline. Sanger validation was not performed.


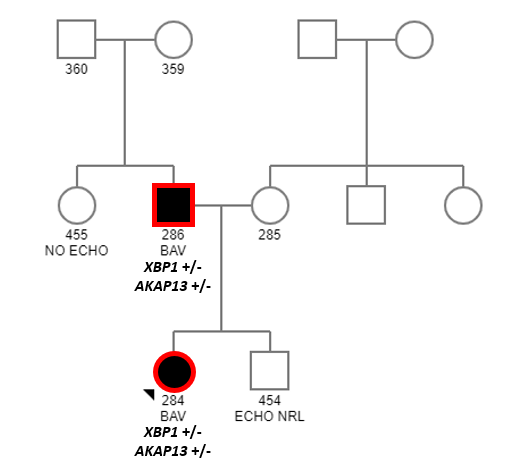


Family 91. *XBP1* and *AKAP13* variants. ES is denoted by red outline. Sanger validation was not performed. ***XBP1*: c.413G>A (p.Gly138Glu). *AKAP13*: c.2510C>T (p.Thr837Met).**


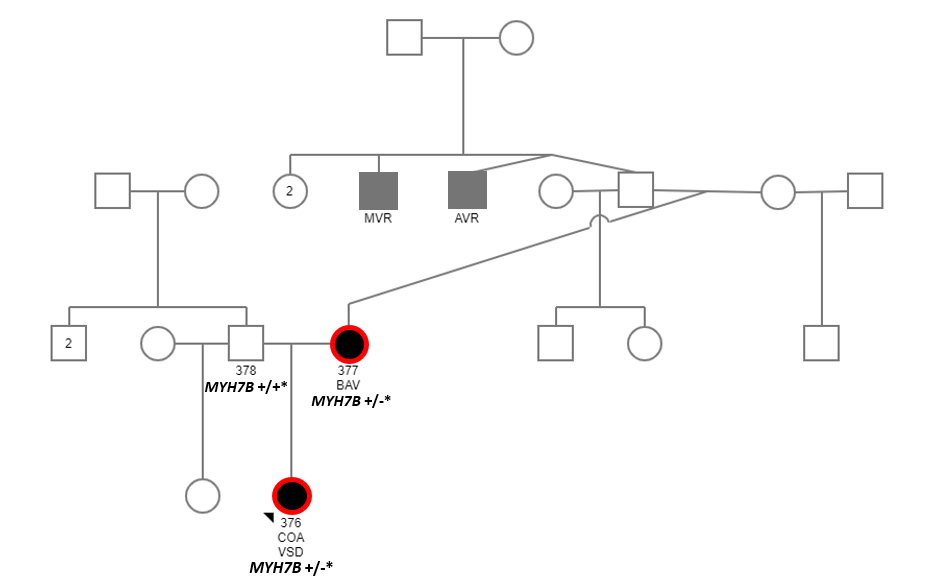


Family 118. *MYH7B*: c.1502T>A (p.Phe501Tyr). ES is denoted by red outline. Sanger validation is denoted as *.


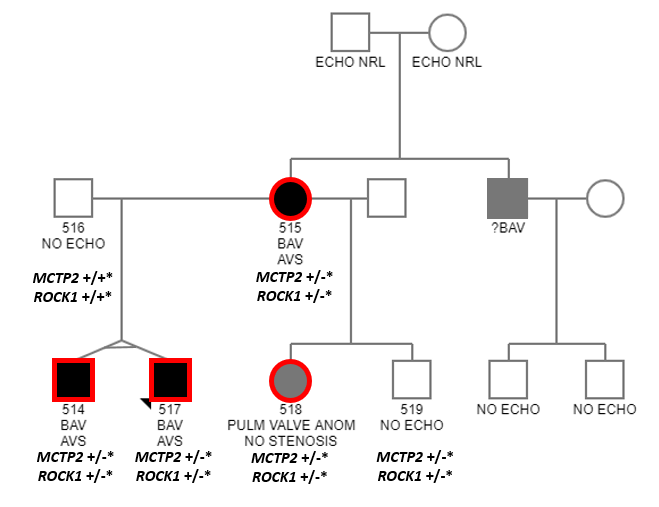


Family 154. *MCTP2* and *ROCK1* variants. ES is denoted by red outline. Sanger validation of variant is denoted by - (reference sequence) or + (variant confirmed). ***MCTP2*: c.65A>C (p.Asn22Thr). *ROCK1*: c.2083A>T (p.Lys695*).** Sanger validation of variant is denoted by - (reference sequence) or + (variant confirmed).


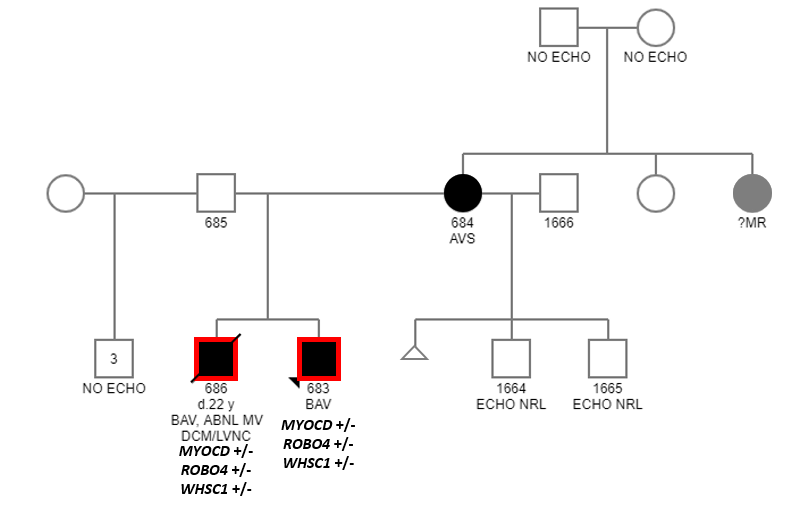


Family 207. *MYOCD*, *ROBO4* and *WHSC1* variants. ES is denoted by red outline. Sanger validation was not performed. ***MYOCD*: c.2452G>A (p.Gly818Arg). *ROBO4*: c.1087G>C (p.Val363Leu). *WHSC1*: c.1910C>T (p.Ser637Leu).**


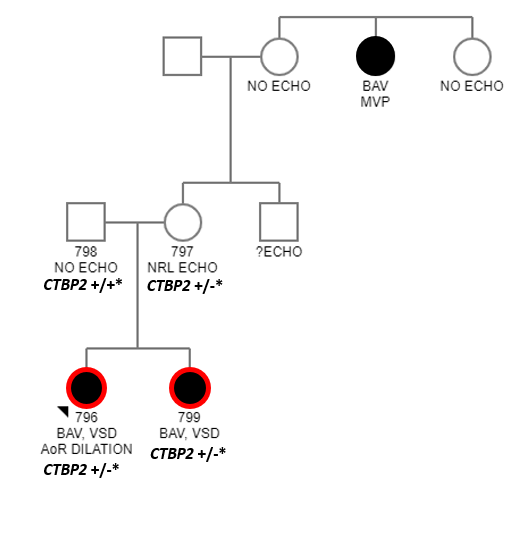


Family 238. *CTBP2*: c.536G>A (p.Arg719His). ES is denoted by red outline. Sanger validation is denoted as*.


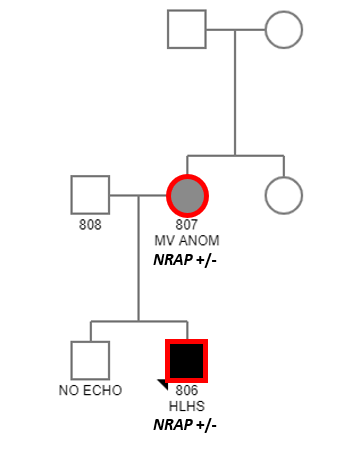


Family 241. *NRAP*: c.4648C>T (p.Arg1550Trp). ES is denoted by red outline. Sanger validation was not performed.


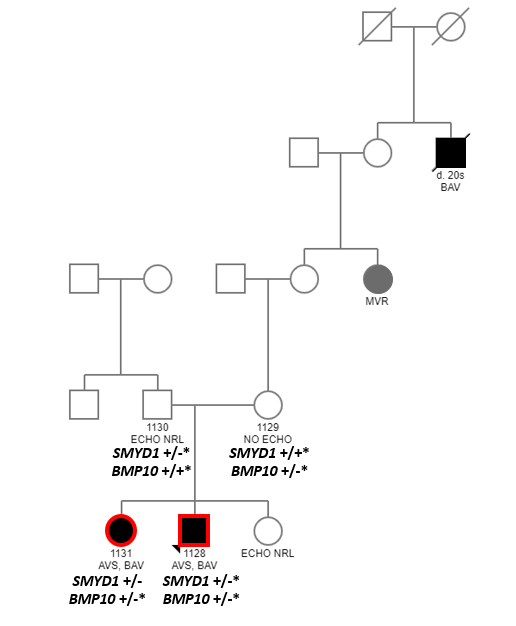


Family 346. *SMYD1* and BMP10 variants. ES is denoted by red outline. Sanger validation is denoted by *. Sanger validation of the *SMYD1* variant was not performed in individual 1131 due to insufficient DNA. ***SMYD1*: c.1321C>T (p.Arg441Trp). *BMP10:* c.625C>T (p.Arg209Cys).**


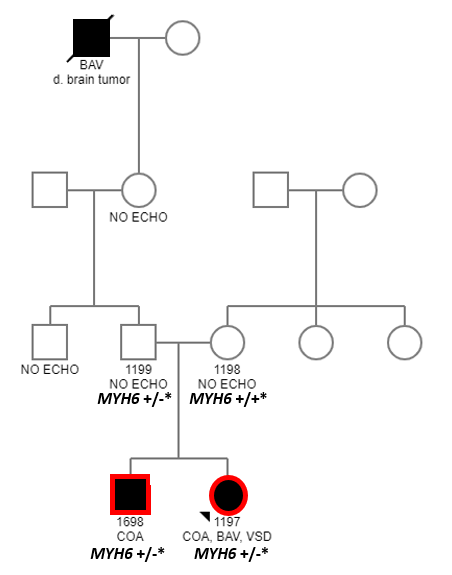


Family 368. *MYH6*: c.734T>C (p.Phe245Ser). ES is denoted by red outline. Sanger validation is denoted by *.


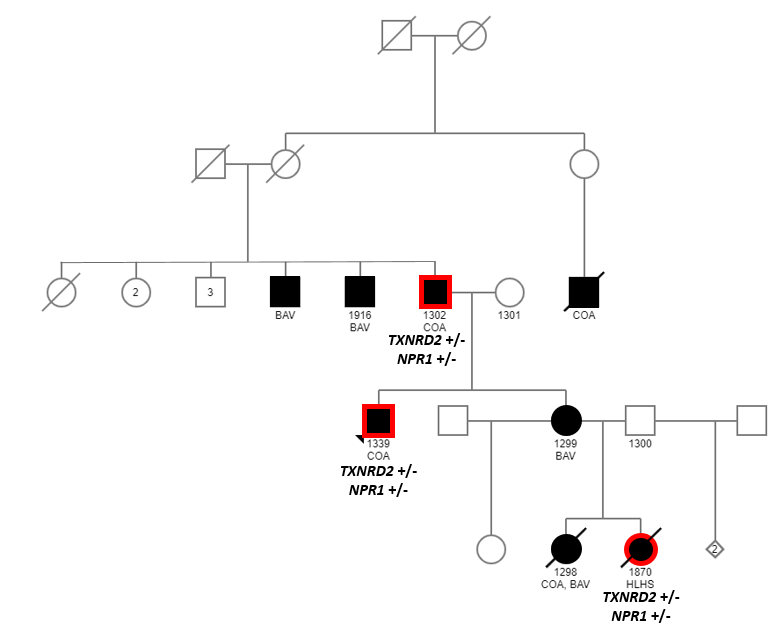


**Family 400. *TXNRD2* and *NPR1* variants**. ES is denoted by red outline. Sanger validation was not performed. ***TXNRD2*: c.591+1G>C. *NPR1*: c.2899G>A (p.Glu967Lys).**


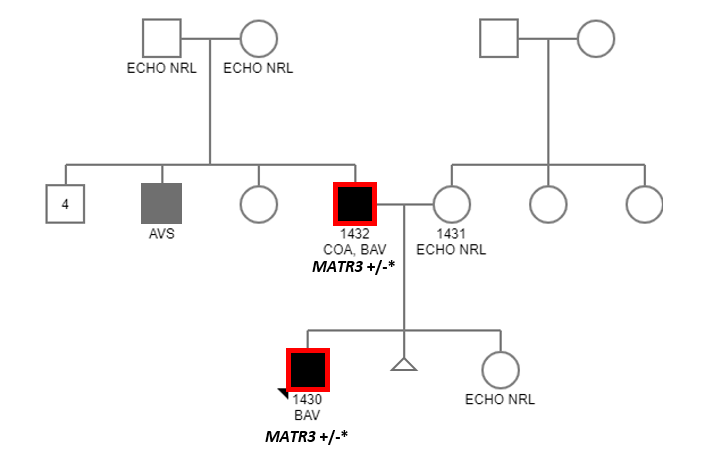


Family 439. *MATR3*: c.629A>T (p.Glu210Val). ES is denoted by red outline. Sanger validation is denoted as *.


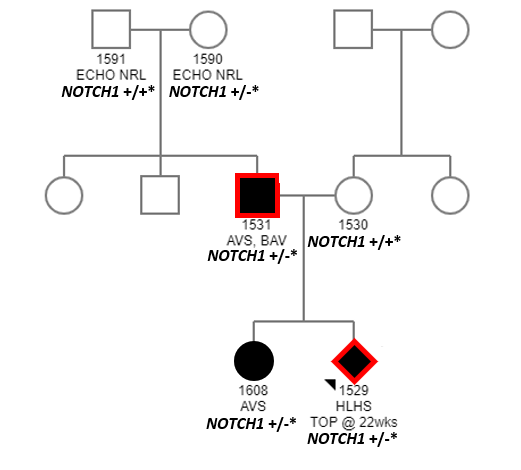


Family 469. *NOTCH1*: c.2995G>A (p.Val999Met). ES is denoted by red outline. Sanger validation is denoted as *.


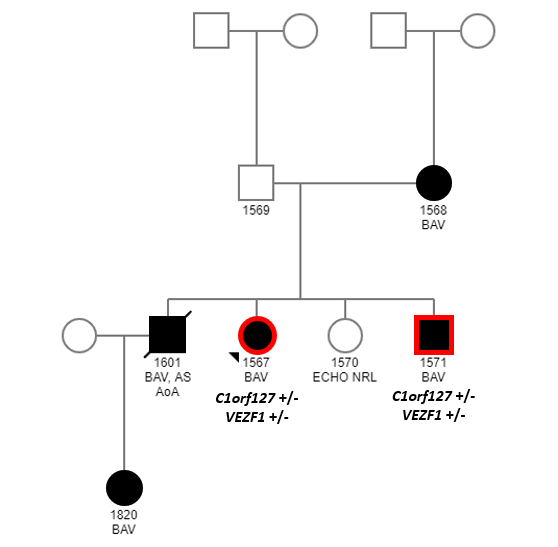


Family 481. *C1orf127* and *VEZF1* variants. ES is denoted by red outline. Sanger validation was not performed. ***C1orf127*: c.516G>C (p.Gln172His). *VEZF1*: c.1041_1042delGC (p.Gln348fs).**


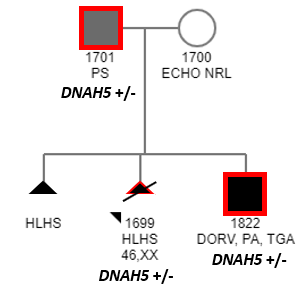


Family 512. *DNAH5*: c.1715T>G (p.Leu572Trp). ES is denoted by red outline. Sanger validation was not performed.


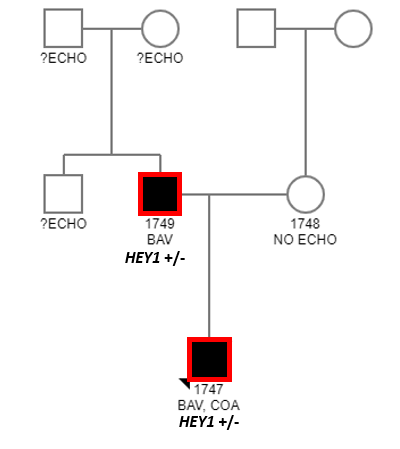


Family 528. *HEY1*: c.800C>A (p.Ser267Tyr). ES is denoted by red outline. Sanger validation was not performed.


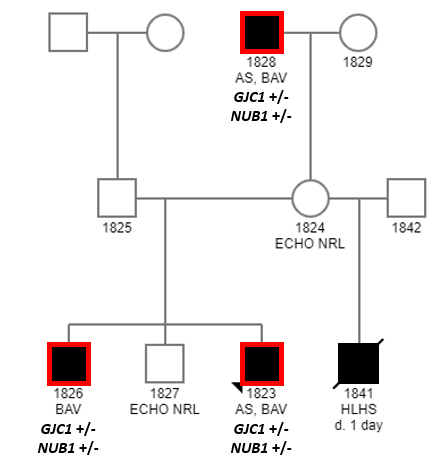


Family 549. *GJC1* and *NUB1* variants. ES is denoted by red outline. Sanger validation was not performed. ***GJC1:* c.928G>A (p.Ala310Thr). *NUB1:* c.84G>T (p.Lys28Asn).**


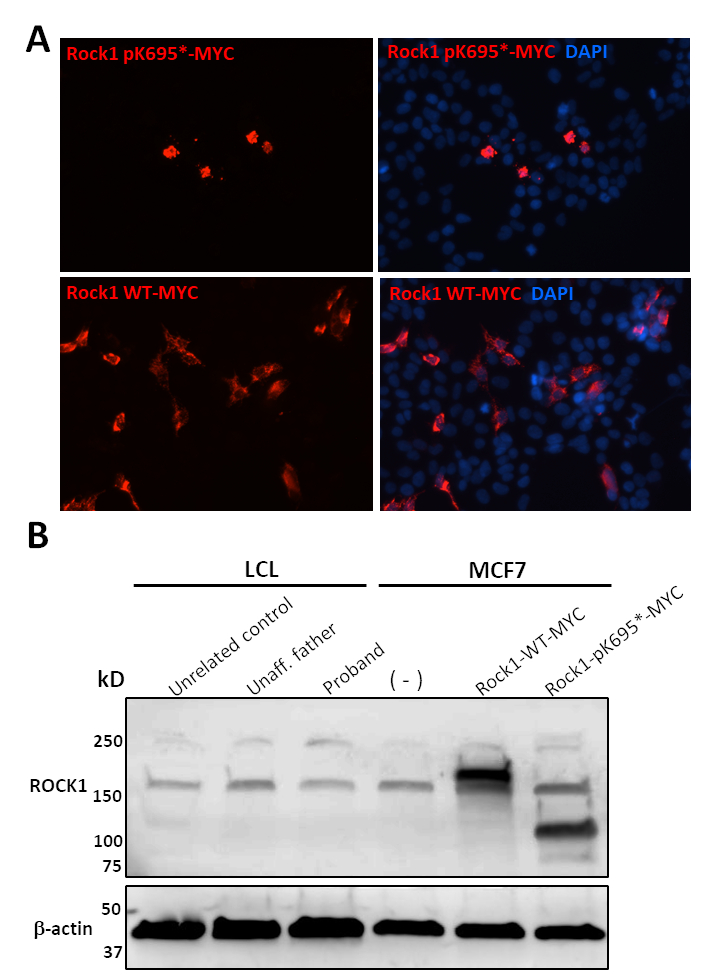


Figure B. Rock1 p695* causes blebbing in MCF7 cells. **A.** MCF7 cells were transfected with either Rock1 p.K695*-MYC or Rock1 WT-MYC expression constructs and stained for the MYC tag after 13 h. Cells expressing the pK695* variant displayed a more compact, globular morphology than the WT-expressing cells, consistent with membrane blebbing associated with activated Rock1. **B.** No signal was detected at the expected size for Rock1 p.K695* in protein from LCLs derived from the proband heterozygous for the ROCK1 c2083A>T variant. In MCF7 cells transfected with MYC-tagged WT and pK695* Rock1 expression constructs, bands were detected at the expected sizes above and below endogenous Rock1.


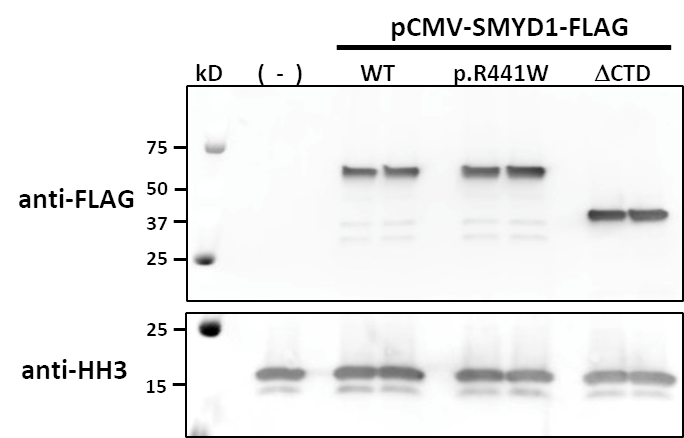


Figure C. FLAG-tagged SMYD1 WT, SMYD1 p.R441W, and SMYD1 ΔCTD were detected at equivalent levels by western blot. Whole-cell extracts (20 μg/lane) from transfected and untransfected (-) HEK293T cells were analyzed in duplicate. The anti-FLAG antibody detected the expected 58 kD band in WT and p.R441W samples, and 35 kD band in the ΔCTD sample. The same blot was reprobed for histone H3 (HH3) as an endogenous reference.


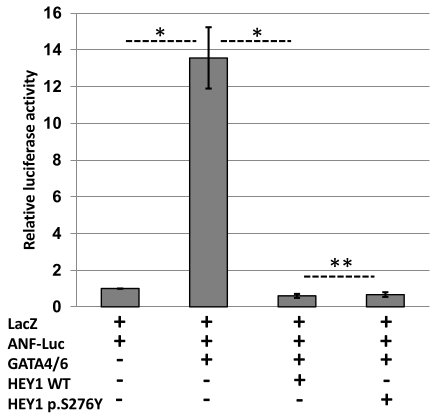


Figure D. HEY1 p.S276Y and HEY1 WT showed the same level of repression of GATA4/6-induced expression from the ANF promoter in a luciferase reporter assay. HEK293T cells were co-transfected with a reporter plasmid that includes the luciferase gene under the control of the ANF promoter and pCMV-LacZ, as a control for transfection efficiency. Co-transfection of constructs that express GATA4 and GATA6 increased luciferase activity by greater than 10-fold. Co-transfection of a HEY1 WT expression construct completely abrogated the effect of GATA4/6 on luciferase activity. Co-transfection of a HEY1 p.S276Y expression construct resulted in repression of luciferase activity that was not significantly different from HEY1 WT. Graphed values represent luciferase activity relative to LacZ activity, normalized to the ANF-Luc control samples. Each bar represents the combined results from three independent experiments with triplicate samples in each experiment. Error bars represent the standard deviation. * P < 0.00001, ** P = 0.246.

# Table A. Sequencing information by subject.

| **Family** | **Subject** | **Sequencing Type** |
| --- | --- | --- |
| **16** | 38 | BHC |
|  | 39 | BHC |
| **19** | 54 | BHC |
|  | 55 | BHC |
|  | 67 | BHC |
| **58** | 160 | BHC |
|  | 162 | BHC |
|  | 168 | BHC |
| **72** | 196 | BHC |
|  | 199 | BHC |
| **91** | 284 | BHC |
|  | 286 | BHC |
| **118** | 376 | BHC |
|  | 377 | BHC |
| **154** | 514 | BHC |
|  | 515 | BHC |
|  | 517 | BHC |
|  | 518 | BHC |
| **207** | 683 | BHC |
|  | 686 | BHC |
| **238** | 796 | BHC |
|  | 799 | BHC |
| **241** | 806 | BHC |
|  | 807 | BHC |
| **346** | 1128 | BHC |
|  | 1131 | BHC |
| **Family** | **Subject** | **Sequencing Type** |
| **368** | 1197 | BHC |
|  | 1698 | BHC |
| **400** | 1301 | NCH2500 |
|  | 1302 | BHC, NCH2500 |
|  | 1339 | BHC, NCH2500 |
|  | 1870 | BHC |
| **439** | 1430 | BHC |
|  | 1432 | BHC |
| **469** | 1529 | BHC |
|  | 1531 | BHC |
|  | 1608 | NCH4000 |
| **481** | 1567 | BHC |
|  | 1569 | NCH4000 |
|  | 1571 | BHC |
|  | 1601 | NCH4000 |
| **512** | 1699 | BHC |
|  | 1701 | BHC |
|  | 1822 | BHC |
| **528** | 1747 | BHC, NCH2500 |
|  | 1748 | NCH2500 |
|  | 1749 | BHC, NCH2500 |
| **549** | 1823 | BHC |
|  | 1826 | BHC |
|  | 1826 | BHC |

Abbreviations: **BHC,** Baylor-Hopkins Center for Mendelian Genomics (HiSeq 2000/2500, HGSC Core, 2x100bp); **NCH2500,** Nationwide Children’s Hospital Biomedical Genomics Core (HiSeq 2500, Agilent Clinical Resource Exome Kit v1, 2x96bp); **NCH4000,** Nationwide Children’s Hospital Biomedical Genomics Core (HiSeq 4000, Agilent Clinical Resource Exome Kit v1, 2x150bp)

Table B. Summary table of rare damaging variants. Genes with rare, damaging variants identified by ES in multiple affected members within 19 families with LVOT defects. References that show association between the gene and congenital heart disease (CHD), as well as reports of a relevant heart phenotype in model organisms are listed on the right. Numbers of individuals with variants in cases and controls in the PCGC dataset are noted in the two far right columns, as numbers of recessive, loss of function and de novo variants separated by “/” (cases n=2871, controls n=1789).

| **Family** | **Phenotype** | **Gene: variant** | **MAF** | **CADD** | **REVEL** | **GERP++** | **Polyphen2 HVar** | **CHD** | **Model** | **PCGC Cases** | **PCGC Controls** |
| --- | --- | --- | --- | --- | --- | --- | --- | --- | --- | --- | --- |
| **16** | HLHS, BAV | ***CASZ1***: NM_001079843.2(CASZ1):c.73C>T:p.Arg25Cys | 0.0007 | 32.0 | 0.47 | 4.5 | 0.99 | 1, 2, 3, 4 | 5, 6 | 0/1/4 | 0/0/0 |
|  |  | ***ELN***: NM_000501.3(ELN):c.2132G>A:p.Gly711Asp | 0.006 | 21.5 | 0.97 | 2.6 | 0.88 | 1 | 7 | 0/5/2 | 0/1/0 |
| **19** | BAV (2), COA/BAV | ***KCNJ2***: NM_000891.2(KCNJ2):c.863A>T:p.Asp288Val | 0 | 24.2 | 0.98 | 5.8 | 0.20 | 30 |  | 0/1/0 | 0/0/0 |
|  |  | ***AKAP13***: NM_007200.4(AKAP13):c.4594G>A:p.Asp1532Asn | 0.0001 | 32.0 | 0.24 | 6.2 | 0.22 | 1 | 8 | 0/0/3 | 1/0/1 |
|  |  | ***CHD8***: NM_001170629.1(CHD8):c.7258C>T:p.Arg2420Cys | 0.0001 | 32.0 | 0.52 | 4.8 | 0.99 | 1 |  | 1/1/0 | 0/0/0 |
|  |  | ***CNTRL***: NM_007018.4(CNTRL):c.38C>A:p.Ala13Glu | 0.0037 | 21.7 | 0.05 | 5.9 | 0.08 | 1 | 9 | 0/4/1 | 0/6/0 |
| **58** | HLHS, BAV, AL | ***AGR3***: NM_176813.4(AGR3):c.400G>T:p.Gly134* | 0.0003 | 40.0 | No Score | 4.3 | No Score |  |  | 0/0/0 | 0/0/0 |
|  |  | ***CLDN20***: NM_001001346.3(CLDN20):c.316C>T:p.Arg106Cys | 0.0003 | 34.0 | 0.54 | 5.4 | 0.91 |  |  | 0/0/0 | 0/0/0 |
|  |  | ***SHF***: NM_001301168.1(SHF):c.527C>T:p.Pro176Leu | 0.0076 | 32.0 | 0.43 | 5.9 | 0.89 | 1 |  | 0/1/0 | 0/1/0 |
|  |  | ***MYOM2***: NM_003970.3(MYOM2):c.1355C>T:p.Ser452Phe | 0.0022 | 27.2 | 0.55 | 4.7 | 0.98 | 1, 31 |  | 0/8/2 | 0/4/1 |
| **72** | AVS/BAV, BAV | ***KRIT1***: NM_194454.1(KRIT1):c.499C>T:p.Arg167Cys | 0.00005 | 32.0 | 0.39 | 4.3 | 0.55 |  |  | 0/0/0 | 0/0/0 |
| **91** | BAV (2) | ***XBP1***: NM_001079539.1(XBP1):c.413G>A:p.Gly138Glu | 0 | 33.0 | 0.30 | 5.9 | 0.99 |  |  | 0/0/0 | 0/0/0 |
|  |  | ***AKAP13***: NM_007200.4(AKAP13):c.2510C>T:p.Thr837Met | 0.0089 | 22.7 | 0.16 | 1.7 | 0.56 | 1 | 8 | 0/0/3 | 1/0/1 |
| **118** | COA, BAV | ***MYH7B***: NM_020884.4(MYH7B):c.1502T>A:p.Phe501Tyr | 0.014 * | 28.1 | 0.71 | 3.5 | 0.99 | 1 |  | 0/7/1 | 1/5/1 |
| **154** | AVS/BAV (3), PVS | ***ROCK1***: NM_005406.2(ROCK1):c.2083A>T:p.Lys695* | 0 | 40.0 | No Score | 4.0 | No Score | 1, 10 | 28 | 0/0/1 | 0/0/0 |
|  |  | ***MCTP2***: NM_018349.3(MCTP2):c.65A>C:p.Asn22Thr | 0.0005 | 32.7 | 0.31 | 3.9 | 0.99 | 1, 11 | 11 | 0/3/0 | 0/2/0 |
| **207** | BAV, BAV/LVNC/AMVT | ***MYOCD***: NM_001146312.2(MYOCD):c.2596G>A:p.Gly866Arg | 0.0002 | 28.1 | 0.23 | 5.1 | 0.85 |  |  | 0/0/0 | 0/0/0 |
|  |  | ***ROBO4***: NM_019055.5(ROBO4):c.1087G>C:p.Val363Leu | 0.0002 | 22.5 | 0.10 | -2.0 | 0.24 | 12 | 12 | 0/0/0 | 1/0/0 |
|  |  | ***NSD2***: NM_001042424.2(NSD2):c.1910C>T:p.Ser637Leu | 0.001 | 22.3 | 0.20 | 2.5 | 0.00 |  | 13 | 0/0/0 | 0/0/0 |
| **238** | BAV/VSD/ARD, BAV/VSD | ***CTBP2***: NM_022802.2(CTBP2):c.2156G>A:p.Arg719His | 0.00003 | 27.8 | 0.74 | 4.7 | 0.30 | 1 | 14 | 0/1/1 | 0/0/0 |
| **241** | HLHS, MVS | ***NRAP***: NM_198060.3(NRAP):c.4648C>T:p.Arg1550Trp | 0.0019 | 34.0 | 0.17 | 4.7 | 0.98 | 1, 15 |  | 0/8/0 | 0/3/0 |
| **346** | AVS/BAV, AVS/BAV | ***SMYD1***: NM_198274.3(SMYD1):c.1321C>T:p.Arg441Trp | 0.014 * | 33.0 | 0.44 | 3.3 | 0.99 | 1 | 16 | 0/1/0 | 0/0/0 |
|  |  | ***BMP10***: NM_014482.1(BMP10):c.625C>T:p.Arg209Cys | 0.012 * | 26.1 | 0.43 | 4.6 | 0.62 | 1 | 17 | 0/1/0 | 0/1/0 |
| **368** | BAV/COA/PDA/VSD, COA/PDA | ***MYH6***: NM_002471.3(MYH6):c.733_734delinsCC:p.Phe245Pro | 0 | No Score | No Score | No Score | No Score | 1,18,19 | 20 | 7/1/4 | 0/2/2 |
| **400** | COA (2), HLHS | ***TXNRD2***: NM_006440.4(TXNRD2):c.591+1G>C | 0.0003 | 26.4 | No Score | 4.6 | No Score |  | 21 | 0/0/0 | 0/1/0 |
|  |  | ***NPR1***: NM_000906.3(NPR1):c.2899G>A:p.Glu967Lys | 0.01 * | 24.8 | 0.14 | 2.9 | 0.24 | 1 |  | 0/1/0 | 0/0/0 |
| **439** | AVS/BAV (2) | ***MATR3***: NM_018834.5(MATR3):c.629A>T:p.Glu210Val | 0.000009 | 23.0 | 0.37 | 5.5 | 0.84 | 22 | 22 | 0/0/0 | 0/0/0 |
| **469** | HLHS, BAV | ***NOTCH1***: NM_017617.4(NOTCH1):c.2995G>A:p.Val999Met | 0.0003 | 24.3 | 0.50 | 4.9 | 0.63 | 1,23,24,25 |  | 2/6/6 | 0/0/0 |
| **481** | BAV (2) | ***C1orf127***: NM_001170754.1(C1orf127):c.516G>C:p.Gln172His | 0.000015 | 24.5 | 0.16 | 4.4 | 0.98 | 1 |  | 0/3/0 | 0/1/0 |
| **512** | HLHS, CT, DORV/dTGA/PVA | ***DNAH5***: NM_001369.2(DNAH5):c.1715T>G:p.Leu572Trp | 0.0013 | 27.7 | 0.80 | 4.9 | 0.85 | 1,10 | 29 | 3/12/1 | 0/4/1 |
| **528** | BAV/COA, BAV | ***HEY1***: NM_012258.3(HEY1):c.800C>A:p.Ser267Tyr | 0.0001 | 28.7 | 0.26 | 5.7 | 0.57 |  |  | 0/0/0 | 0/0/0 |
| **549** | AVS/BAV (2), BAV | ***GJC1***: NM_005497.3(GJC1):c.928G>A:p.Ala310Thr | 0.0027 | 26.1 | 0.51 | 5.5 | 0.18 | 10 | 27 | 0/0/0 | 0/0/0 |
|  |  | ***NUB1***: NM_001243351.1(NUB1):c.84G>T:p.Lys28Asn | 0.017 * | 23.2 | 0.11 | 5.6 | 0.76 | 1 |  | 0/0/1 | 0/4/0 |

Abbreviations: **AMVT**, Accessory mitral valve tissue; **ARD**, Aortic root dilation; **AL**, Arteria lusoria; **AVS**, Aortic valve stenosis; **BAV**, Bicuspid aortic valve; **CADD**, combined annotation-dependent depletion (ref, PMID: 30371827); **COA,** Coarctation of the aorta; **CT**, Cor triatriatum; **DORV/dTGA**, Double outlet right ventricle/dextro-transposition of the great arteries; **HLHS**, Hypoplastic left heart syndrome; **LVNC**, Left ventricular noncompaction; **MAF**, Maximum minor allele frequency across populations in gnomAD 3.0 genomes and exomes; **MVS**, Mitral valve stenosis; **PDA**, Patent ductus arteriosus; **PVA**, Pulmonary valve atresia; **PVS**, Pulmonary valve stenosis; **VSD**, Ventricular septal defect.

* MAF > 0.01 due to gnomAD 3.0 update

**References cited in Table B:**

| 1 | Jin *et al.*, 2017,PMID: 28991257 |
| --- | --- |
| 2 | Guo *et al.*,2019, PMID:31268246; |
| 3 | Huang *et al.*, 2016, PMID:27693370; |
| 4 | Qiu *et al.*, 2017,PMID: 28099117 |
| 5 | Dorr *et al.*, 2015, PMID:25953344 |
| 6 | Liu *et al.*, 2014, PMID:25190801 |
| 7 | Krishnamurthy *et al.*, 2012, PMID: 22265892 |
| 8 | Mayers *et al.*, 2010, PMID: 20139090 |
| 9 | Lo. 2011. MGI Direct Data Submission J:175213 |
| 10 | Li *et al.*, 2017, PMID: 29089047 |
| 11 | Lalani *et al.*, 2013,PMID: 23773997 |
| 12 | Gould *et al.*, 2019, PMID: 30455415 |
| 13 | Nimura et al., 2009, PMID: 19483677 |
| 14 | Hildebrand and Soriano, 2002,PMID: 12101226 |
| 15 | Truszkowska *et al.*, 2017, PMID:28611399 |
| 16 | Gottlieb *et al.*, 2002 PMID:11923873 |
| 17 | Chen *et al.*, 2004, PMID: 15073151 |
| 18 | Theis *et al.*, 2015, PMID: 26085007 |
| 19 | Liu *et al.*, 2020, PMID: 33131162 |
| 20 | Jones *et al.*, 1996, PMID: 8878443 |
| 21 | Conrad *et al.*, 2004, PMID:15485910 |
| 22 | Quintero-Rivera *et al.*, 2015, PMID:25574029 |
| 23 | Garg *et al.*, 2005,PMID: 16025100 |
| 24 | McBride *et al.*, 2008, PMID: 18593716; |
| 25 | Kerstjens-Frederikse *et al.*, 2016 PMID: 26820064; |
| 26 | Koenig *et al.*, 2016, PMID: 27107132 |
| 27 | Kumai *et al.*, 2000, PMID:10903175 |
| 28 | Phillips *et al.*, 2013,PMID: 23723064 |
| 29 | Tan *et al.*, 2007, PMID: 18037990 |
| 30 | Andelfinger et al 2002, PMID: 12148092 |
| 31 | Auxerre-Plantié E, et al. 2020, PMID: 33033063 |

Table C. RNA-Seq analysis in Family 154. RNA-Seq analysis for the presence of the *ROCK1* c.2083T>A variant in RNA from LCLs derived from the proband (517) and his unaffected father (516) from family 154, and an unrelated control (5698).

| **Sample** | **Reads** | **Base*** | **Count** | **% of Total** |
| --- | --- | --- | --- | --- |
| Affected Proband (517) | 227 | A | 38 | 17 |
|  |  | C | 0 | 0 |
|  |  | G | 0 | 0 |
|  |  | T | 189 | 83 |
|  |  | N | 0 | 0 |
| Unaffected Father (516) | 385 | A | 0 | 0 |
|  |  | C | 0 | 0 |
|  |  | G | 0 | 0 |
|  |  | T | 385 | 100 |
|  |  | N | 0 | 0 |
| Control (5698) | 294 | A | 0 | 0 |
|  |  | C | 0 | 0 |
|  |  | G | 0 | 0 |
|  |  | T | 294 | 100 |
|  |  | N | 0 | 0 |

*Chr18:20991236 (Reference base T)

# Supporting Results

## ROBO4 p.Val363Leu Variant in Familial BAV

Family 207 has three members with LVOT defects, one with BAV, one with BAV and a dysplastic mitral valve, and one with AVS (**Figure A**). ES was performed on the two individuals with BAV. Of the 77 filtered variants, three candidates were identified: *MYOCD* (NM_001146312.2:c.2596G>A, p.Gly866Arg), *NSD2* (NM_001042424.2:c.1910C>T, p.Ser637Leu), and *ROBO4* (NM_019055.5:c.1087G>C, p.Val363Leu), which was considered the most likely candidate.

ROBO4 (Roundabout Guidance Receptor 4) is a receptor for Slit proteins and has recently been shown to be involved in angiogenesis and vascular patterning.[1] The *ROBO4* c.1087G>C variant has a maximum population allele frequency of 0.00006 in gnomAD and was predicted to be damaging by CADD, SIFT, and PolyPhen2 Complex. ACMG criteria calls this a VUS; presence in six individuals in gnomAD (one above the cut off five for PM2/BS2 criteria) reduced this from likely pathogenic. Given the presence of reduced penetrance in CHD, we would consider this criterion not as relevant in this instance. *ROBO4* disease causing variants were identified in two families with BAV and thoracic aortic aneurysm (TAA) and was also found in 1.8% of a cohort of individuals with BAV and TAA.[1] ROBO4 mutant zebrafish have altered flow across the ventriculo-bulbar valve, while knockout mice show a reduced penetrance aortic valve phenotype of BAV, stenosis, regurgitation, and ascending aorta dilation.[1] Functional testing was not performed on the ROBO4 variant given the evidence it is a CHD causing gene.

## CTBP2 p.Arg719His Variant in Familial BAV/VSD

Family 238 has three members with BAV, of which two also have VSD and one has mitral valve prolapse (**Figure A**). In ES data from two affected members of family 238, 94 variants remained after filtering, of which a nonsynonymous SNV in *CTBP2* (NM_022802.2:c.2156G>A, p.Arg719His) was the most likely candidate as a contributing factor to the CHD.

CTBP2 (C-Terminal Binding Protein 2) is an NADH-dependent transcriptional corepressor that is recruited to gene regulatory sites by DNA-binding transcription factors, where it mediates transcriptional repression through binding of histone deacetylases and histone methyltransferases.[2] In a knockout mouse, loss of CtBP2 results in embryonic lethality at E10.5, with multiple abnormalities that include defects in heart morphogenesis.[3] A study of CNVs in CHD patients identified a *de novo* 10q25-26 deletion that encompasses *CTBP2,* with a novel p.Arg134Trp variant in the remaining *CTBP2* allele in a patient with TA and right aortic arch.[4] CHD is found in ~40% of patients with 10q25 and 10q26 deletions.[5] Frameshift variants in *CTBP2* were recently reported in two patients with LVOT defects.[6] The variant we identified in *CTBP2* lies within the NAD+ binding domain. In *CTBP2* transcript NM_022802.2, formerly known as protein RIBEYE, the same changes take place at nucleotide 2156 (residue 719). The maximum population frequency of this variant is 0.00003, and it is predicted to be deleterious by CADD, SIFT, GERP++, and Polyphen2 Complex. ACMG criteria define this variant as likely pathogenic. Functional testing not performed on the CTBP2 variant given the evidence it is a CHD causing gene.

## MYH6 p.Phe245Pro Variant in Familial COA

Family 368 (**Figure A**) has two siblings with COA, with the proband also having BAV and VSD, and paternal great grandfather with BAV (not sequenced). Of 96 variants in family 368 that passed filtering, a two-base block substitution variant in *MYH6* was the strongest candidate to be a contributing factor.

*MYH6* (Myosin Heavy Chain 6) encodes α-cardiac myosin heavy chain, a major structural component of cardiac muscle thick filaments. In previous pedigree studies, damaging variants in *MYH6* were found to be associated with a range of CHDs, including ASD,[7] tricuspid atresia, VSD, TGA, and AVS.[8] In a large-scale ES study of CHD trios, potentially damaging recessive and *de novo* variants in *MYH6* were found in 10 probands with LVOT defects, TGA and CTD.[6] The two-base *MYH6* substitution in family 368 (NM_002471.3:c.733_734delinsCC, p.Phe245Pro) causes a substitution of proline for phenylalanine at residue 245. Both affected members of family 368 are heterozygous for the paternally inherited variant, where the father had not undergone an echocardiogram. The variant is located two bases from the end of exon 8, potentially affecting splicing, at a position that is highly conserved in vertebrates. The block substitution is not found in gnomAD, but the maximum population frequency of either single-base change is 0.000077 in gnomAD, and both single-base changes are predicted to be deleterious by CADD, SIFT, GERP++, Polyphen2 Complex and Mendelian, MetaSVM, and MetaLR. There is one report of p.Phe245Leu in ClinVar , listed as a pathogenic variant for familial dilated cardiomyopathy (accession RCV000624469.1). ACMG criteria call this variant as pathogenic. Because it is established that variants in *MYH6* contribute to CHD, functional analysis was not performed on this variant.

## MATR3 p.Glu210Val Variant in Familial AVS/BAV

Family 439 has one member with BAV and one with BAV and COA (**Figure A**). Filtering of variants from the two affected members of family 439 yielded 94 variants, of which a substitution in *MATR3* (NM_018834.5:c.629A>T, p.Glu210Val) was the strongest candidate.

MATR3 (Matrin 3) is a nuclear matrix protein that functions in RNA metabolism, including alternative splicing and polyadenylation and in chromatin remodeling.[9] Loss of Matrin-3 results in interrupted topological associated domain insulation at CTCF sites, affecting cell differentiation.[10] It has been previously associated with motoneuron disorders and distal myopathy.[11, 12] A gene trap mutant allele of mouse *Matr3*, in which a beta geo cassette is inserted into exon 13, is embryonic lethal at or before implantation in homozygotes. Heterozygotes show a range of heart malformations, including VSD, DORV, CoA and BAV, with incomplete penetrance in embryos and newborn pups.[13] Similarly, a patient with a balanced translocation, 46, XY,t(1;5)(p36.11;q31.2) that includes a breakpoint in the 3’ UTR of *MATR3*, was diagnosed with BAV, CoA and PDA.[13] The MATR3 p.Glu210Val variant in family 439 has a maximum population frequency of 0.000008, and it is predicted to be deleterious by CADD, SIFT, GERP++, and Polyphen2 Complex and Mendelian. ACMG criteria call this variant pathogenic. Functional testing of this variant was not performed given the known association with LVOT defects.

## NOTCH1 p.Val999Met Variant in Familial AVS with HLHS and BAV Cases

Family 469 has three family members with LVOT defects, HLHS in the proband BAV and critical AVS in a sister, and BAV in the father (**Figure A**). Of the 35 variants in family 469 that passed filtering, *NOTCH1* p.Val999Met was the top candidate.

NOTCH1 (Notch Receptor 1) is a transmembrane receptor that plays a central role in a highly conserved signaling pathway that regulates cell specification during development. The causative role of NOTCH1 variants in LVOTs is well-established.[14-16] The *NOTCH1* variant (NM_017617.4:c.2995G>A, p.Val999Met) has a maximum population frequency of 0.000048, and is predicted to be deleterious by CADD, SIFT, GERP++, Polyphen2 Complex and Mendelian, MetaSVM, and MetaLR. ACMG criteria call this a VUS; presence in five individuals in gnomAD (at the cut off for PM2/BS2 criteria) reduced this from likely pathogenic. Given the presence of reduced penetrance in CHD, we would consider this criterion not as relevant in this instance. Functional testing was not performed on the NOTCH1 variant given the evidence it is a CHD causing gene.

# Supporting Materials and Methods

## Clinical Evaluation

All families were assessed by a clinical geneticist (KLM) for evidence of other phenotypic features that might suggest additional diagnoses or presence of a syndrome. No families were diagnosed with a specific syndrome.

## Plasmids

The plasmid pCMV-Tag2A-FLAG-CASZ1b [17], containing the full-length coding sequence for the human CASZ1b isoform with an N-terminal FLAG tag was generously provided by Dr. Zhihui Liu and Dr. Carol J. Thiele. The tyrosine hydroxylase-luciferase (TH-Luc) reporter plasmid was designed based on a previous report[18]. A 2 kb sequence immediately upstream of TH exon 1 was PCR amplified from human genomic DNA (GM1200 lymphoblastoid line, Coriell) using PCR primers 5’-TAGGTACCAGGCAAATCCCTCCAACGC-3’ and 5’-TACTCGAGGGCTCAGTGTGGAGGTC-3’ that included an added *Kpn*I and *Xho*I site (underlined) on each primer respectively. After digestion with *Kpn*I and *Xho*I, the PCR product was ligated into the pGL4.10[luc2] vector (Promega, E6651). The pCMV6-Rock1 plasmid, containing the full-length mouse *Rock1* coding sequence was purchased from Origene (MR224143). In order to generate N-terminal-tagged Rock1 and obtain stronger immunostaining signal from the tagged protein, the 4.1 kb *Rock1* coding sequence was transferred from the pCMV6-Entry vector into the pCAG-6xMyc vector. Briefly, pCMV6-Rock1 was digested with *Asi*SI and *Mlu*I, the pCAG-6xMyc vector was digested with *Fse*I and *Asc*I, the overhanging ends were filled in with Klenow fragment, and the 4.1 kb Rock1 fragment was blunt-end ligated into the 5.5 kb linearized pCAG-6xMyc vector using T4 DNA ligase (New England BioLabs, #M0202). An expression construct with the full-length coding sequence for human *BMP10* in the pCMV-AC-GFP expression vector was purchased from Origene (RG215695). The *BMP10* open reading frame was excised and cloned into pcDNA3.1/V5-6xHis (ThermoFisher, V81020). An expression construct that includes the human *SMYD1* coding sequence (NM_198274) in the pCMV6-Entry vector (Origene, RC221269) was purchased from Origene (RC221269). The pCMV-SMYD1-ΔCTD construct was engineered by introducing an *Xho*I site at nucleotide positions 883-888 of the *SMYD1* coding sequence in the expression construct using site-directed mutagenesis with primers SMYD1-XhoI forward and reverse. The plasmid was then digested with *Xho*I, releasing a 650 bp fragment that encodes the C-terminal domain of SMYD1. The remaining vector with the N-terminal region of *SMYD1* was recircularized with T4 DNA ligase to generate a construct that expresses SMYD1 truncated at aa position 294. Full-length human *HEY1* coding sequence was PCR amplified from cDNA synthesized from RNA from RD cells (ATCC, CCL-136). The PCR product was ligated into pcDNA3.1/V5-His. Human *KCNJ2* coding sequence corresponding to GenBank accession number NM_000891.3 was cloned into the pMT3 mammalian expression plasmid.

## Site-directed mutagenesis

Sequence variants for *CASZ1, Rock1, SMYD1*, *BMP10*, and *HEY1* were introduced into their respective expression constructs using the Q5 Site-Directed Mutagenesis Kit (New England BioLabs, E05545) according to the manufacturer’s protocol. For KCNJ2, the variant was introduced using the Stratagene QuickChange site-directed mutagenesis kit (Cedar Creek, TX). The mutagenic primer pairs used for PCR amplification of the plasmids are listed below. Sanger sequencing of the entire coding sequence in all the modified plasmids was used to verify the presence of the desired variant and absence of any additional sequence changes.

*BMP10* c.625C>T forward 5’-TGCCATCAGA**T**GTTGGCAAAAG-3’

reverse 5’-TCTGTGACATCAAAAGTCTC-3’

*CASZ1* c.73C>T forward 5’-GGCGCCCAAA**T**GCAAGGGTGG-3’

reverse 5’-ATGGCGGGCTTGCCTGCA-3’

*HEY1* c.800C>A forward 5’-TTCCCCTTCT**A**TTTCGGCTCCTTCC-3’

reverse 5’- GGCCGACAGGGAGGCCAC-3’

*Rock1* c.2083A>T forward 5’-TAAAGTCACC**T**AAGCACGCCT-3’

reverse 5’-TGTTCATTTACTTCTTGTTCTAAC-3’

*SMYD1* c.1321C>T forward 5’-AGAGGCCATG**T**GGGTGCAGAC-3’

reverse 5’-AAGTCCTTAGTGATGGGGTGG-3’

*SMYD1*-XhoI forward 5’-AAAGACAACC**TCGA**GCCCTCTCAG-3’

reverse 5’-CACCCCCAGGAAGAGGTC-3’

## Western Blotting

Unless otherwise noted, whole-cell protein extracts were prepared by rinsing cells with ice-cold PBS, followed by lysis in RIPA lysis buffer (150 mM NaCl, 1 % v/v NP-40, 0.5% w/v sodium deoxycholate, 0.1% SDS, 25 mM Tris, pH 7.4) supplemented with HALT Protease and Phosphatase Inhibitor Cocktail (ThermoFisher Scientific, 78446). Protein concentrations were measured using a BCA Protein Assay kit (Pierce, 23227) and a Nanodrop 2000 spectrophotometer (ThermoFisher Scientific). Protein extracts were resolved by SDS-PAGE on 4-15% polyacrylamide gradient gels (Mini-Protean TGX, Bio-Rad, 4561083) and transferred to PVDF membrane (Amersham HyBond, GE Healthcare Life Sciences, 10600023). After probing the blots with primary antibody, blots were incubated with AP-conjugated secondary antibodies (donkey anti-rabbit IgG-AP, Jackson ImmunoResearch, 711-055-152; goat anti-mouse IgG-AP, Cell Signaling Tech., 7056) and signal was visualized by incubating for 5 min with ECF Substrate (GE Healthcare Life Sciences, RPN5785). Fluorescent signal was captured on a Typhoon FLA 9500 imager (GE Healthcare Life Sciences). Western blot signal was quantitated using ImageQuant TL software (GE Healthcare Life Sciences).

## Antibodies

Primary antibodies: anti-FLAG mouse monoclonal (clone M2, Sigma, F1804), anti-Myc-tag mouse monoclonal (clone 9E10, Origene, TA150121), anti-β-Tubulin mouse monoclonal (clone A-6, Santa Cruz Biotechnology, SC-398103), anti-Actin mouse monoclonal (clone C4, Abcam, ab3280), anti-ROCK1 N-terminus, rabbit polyclonal (GeneTex, GTX113266), anti-Histone H3 rabbit polyclonal (GenScript, A01502), anti-BMP10 rabbit polyclonal (ThermoFisher, PA5-11711), anti-BMP10 growth factor domain (aa 360-424) (Invitrogen, PA5-21940).

## Immunofluorescence microscopy

For analysis of CASZ1 subcellular localization, HEK293T cells (ATCC, CRL-11268) and H9c2 (ATCC, CRL-1446) cells were seeded in 12-well plates at 3x10^5^ and 5x10^4^ cells/well respectively. After 24 h, the cells were transfected with 1 ug/well of either pCMV-CASZ1 WT or pCMV-CASZ1 c.73C>T using Lipofectamine 2000 (Invitrogen, 11668027) according to the manufacturer’s instructions. After 24 h, the cells were fixed with 2% paraformaldehyde and stained with mouse anti-FLAG antibody diluted 1:2000 in blocking solution (5% normal goat serum in PBS). The cells were rinsed and incubated with Alexa Fluor 488-conjugated goat anti-mouse IgG (Invitrogen, #A11001) diluted 1:1000 in blocking solution. To determine the effect of ROCK1 c.2083A>T expression on cell morphology, MCF7 cells were seeded in 12-well plates and after 24 h transfected with pCAG-Rock1 WT or pCAG-Rock1 c.2083A>T at 1 ug/well with Lipofectamine 2000. One day later, the cells were fixed in 2% paraformaldehyde and stained with rabbit anti-c-MYC polyclonal antibody (Santa Cruz Biotechnology, #SC789) at 1:500 dilution in blocking solution followed by staining with Alexa Fluor 594 goat anti-rabbit IgG at 1:1000 dilution. All stained samples were mounted with ProLong Gold antifade reagent with DAPI (Invitrogen, P36935) and a coverslip. Photomicrographs were obtained using an EVOS FL auto imager (Life Technologies).

## Cell fractionation

For analysis of CASZ1 subcellular localization, HEK293T cells were seeded at 2x10^5^ cells/well in a 12-well plate. The following day, triplicate wells were transfected with 1 ug of either pCMV-CASZ1 WT or pCMV-CASZ1 c.73C>T using Lipofectamine 2000. After 24h, the cells were trypsinized (TripLE Express, Gibco, 12605010) and rinsed in PBS, pH7.4. the samples were resuspended in 1 mL PBS, split into two aliquots, and centrifuged at 400 rcf for 2 min. After discarding the supernatant, one aliquot was lysed by adding 100 μL RIPA buffer with 1 mM DTT and 1 mM PMSF, vortexed and placed on ice for 20 min. The other aliquot was resuspended in 100 μL of cytoplasmic extract buffer (10 mM HEPES, 60 mM KCl, 1 mM EDTA, 0.075% v/v NP-40, 1 mM DTT, 1 mM PMSF, pH 7.6) and incubated on ice for 3 min with gentle mixing. Nuclei were pelleted by centrifugation of the lysates at 100 rcf for 2 min. The supernatant (cytoplasmic fraction) was transferred to a clean tube and placed on ice. The nuclear pellet was resuspended and pelleted twice in 200 μL of cytoplasmic extract buffer without NP-40. The supernatant was discarded, and the nuclear pellet was lysed in 200 μL RIPA buffer with 1 mM DTT and 1 mM PMSF. The nuclear lysates were homogenized on ice using a microtip sonicator (Fisherbrand) at amplitude 20 for 20 s. The protein concentration of the lysates was measured and 10 ug of each protein extract was analyzed by western blotting. Western blots were probed with mouse anti-FLAG (1:2000 dilution), mouse anti-α-Tubulin (1:500 dilution) and rabbit anti-Histone H3 (1:10,000 dilution) in blocking solution. After washing, the blots were incubated with AP-conjugated secondary antibodies (Santa Cruz Biotechnology, goat anti-mouse IgG-AP and donkey anti-rabbit IgG-AP, 1:5000 dilution). Signal was visualized using Amersham ECF Substrate and detected using a Typhoon FLA 9500 imager.

## Luciferase assays

For CASZ1 luciferase assays, HEK293T cells were seeded at 10^5^ cells/well in a 24-well plate. The next day, the cells were transfected with combinations of plasmids pCMV-LacZ, pGL4.1-TH-Luc reporter, and either pCMV-CASZ1 WT, pCMV-CAS1 c.73C>T or pCMV-Tag2A (empty vector) using Lipofectamine 2000. Transfection reactions with different combinations of plasmids were supplemented with empty vector DNA such that all transfections included the same amount of total plasmid DNA. For SMYD1 luciferase assays, HEK293T cells were transfected with combinations of pCMV-LacZ, pGL3-promoter (SV40) reporter, and either pCMV6-SMYD1 WT, pCMV6-SMYD1 c.1321C>T or pCMV6-SMYD1 ΔCTD using Lipofectamine 2000, and cell lysates prepared after 24 h. For HEY1 luciferase assays, HeLa cells were seeded in 6-well plates at 5.0x10^5^ cells/well and after 24 h, transfected with pCMV-LacZ, pGL2-ANF, pDEST27-GATA4 and pDEST27-GATA6, and either pcDNA3-HEY1 WT or pcDNA3-HEY1 c.800C>A using FuGENE 6 transfection reagent (Promega, E2691) according to the manufacturer’s instructions. In each transfection, plasmid DNAs were supplemented with empty vector DNA so that all transfections included the same amount of total DNA. In all experiments, luciferase and β-galactosidase expression were assayed 24 h after transfection using an Invitrogen Dual-Light Reporter Gene Assay System (ThermoFisher Scientific, T1003) according to the manufacturer’s instructions. Briefly, cells were rinsed with PBS and 50-100 μL of lysis buffer was added to each well. Cell lysates were transferred to microfuge tubes and centrifuged at 17k rcf for 5 min. The supernatants were loaded into an opaque 96-well plate (10 uL/well) and 25 μL of Buffer A was added to each sample. Luciferase activity was measured using a LUMIstar Omega luminometer (BMG Labtech), that injected 100 μL of Buffer B with Galacton-Plus substrate into each sample and measured luminescence after 1 sec. After 1h, β-galactosidase activity was measured in the same samples by injecting 100 μL of Accelerator-II and measuring luminescence after 1 sec. The luciferase signal was normalized to the β-galactosidase signal in each sample to control for variation in transfection efficiency.

## RNA-Seq

Lymphoblastoid cell lines (LCL) were generated as previously described (Oh *et al.*, 2003) from the proband and his unaffected father from family 154, and an unrelated unaffected individual. Total RNA was isolated from the LCLs using a High Pure RNA isolation kit (Roche, 11828665001), including DNase I treatment, according to the manufacturer’s instructions. Strand-specific RNA-Seq libraries were prepared using NEBNext Ultra II Directional RNA Library Prep Kit for Illumina, following the manufacturer’s recommendations. In summary, total RNA quality was assessed using RNA 6000 Nano kit on Agilent 2100 Bioanalyzer (Agilent Biotechnologies, 5067-1511) and concentration measured using Qubit RNA HS assay kit (Life Technologies, Q32852). A 250-500 ng aliquot of total RNA was rRNA depleted using NEB’s Human/Mouse/Rat RNAse-H based Depletion kit (New England BioLabs, E7400). Following rRNA removal, mRNA was fragmented and then used for first- and second-strand cDNA synthesis with random hexamer primers. ds cDNA fragments underwent end-repair and a-tailing and ligated to dual-unique adapters (Integrated DNA Technologies). Adaptor-ligated cDNA was amplified by limit-cycle PCR. Library quality was analyzed on Tapestation High-Sensitivity D1000 ScreenTape (Agilent Biotechnologies, 5067- 5585) and quantified by KAPA qPCR (KAPA BioSystems). Libraries were pooled and sequenced at 2 x 150 bp read lengths on the Illumina HiSeq 4000 platform. On average, 62 million paired-end 151 bp RNA-Seq reads were generated for each sample (the range was 62 to 70 million). Low-quality reads (q<10), and adaptor sequences were eliminated from raw reads using bbduk version 37.64 (<https://jgi.doe.gov/data-and-tools/bbtools/bb-tools-user-guide/bbduk-guide/>). Each sample was aligned to the GRCh38.p12 assembly of the human reference from NCBI (<https://www.ncbi.nlm.nih.gov/assembly/GCF_000001405.38/>) using version 2.6.0c of the RNA-Seq aligner STAR (<http://bioinformatics.oxfordjournals.org/content/29/1/15>). Transcript features were identified from the Generic Feature Format (GFF) file provided with the assembly from NCBI. The single nucleotide position metrics were generated using bam-readcount (<https://github.com/genome/bam-readcount.git>).

## *Rock1* expression

MCF7 cells (ATCC, HTB-22) were seeded at 6x10^5^ cells/well in a six-well plate and the next day, transfected with either pCAG-Rock1 WT or pCAG-Rock1 c.2083A>T at 3 ug DNA/well using Lipofectamine 2000. After 24 h, whole-cell extracts were prepared in RIPA buffer with HALT protease inhibitor. A western blot was prepared with 20 ug/lane of protein extract from the lymphoblastoid cell lines and Rock1-transfected MCF7 cells. The blot was cut and probed overnight with rabbit anti-Rock1 (N-terminus) antibody (GeneTex, GTX113266) at 1:2000 dilution and mouse anti-α-actin antibody (Abcam, #ab3280) at 1:5000 dilution, followed by donkey anti-rabbit IgG-AP and goat anti-mouse IgG-AP, both at 1:5000 dilution. Signal was visualized by incubating the blot with ECF substrate and detecting fluorescence with a Typhoon 9500 imager.

## *BMP10* Stable Transfectants

HEK293T cells were transfected with the pcDNA3-BMP10 WT or c.625C>T expression vectors using FuGENE 6 transfection reagent following manufacturer’s protocol. Stably transfected cells were selected using Geneticin (ThermoFisher, 10131035) at 500ug/ml, and subcultured into monoclonal cell lines. The relative level of *BMP10* expression was measured in a series of stable transfectants by qRT-PCR. cDNA was synthesized directly from individual lines, untransfected HEK293T cells (negative control) and cells transiently transfected with pcDNA3-BMP10 WT (positive control) using the FastLane Cell cDNA kit (Qiagen, 215011). PCR reactions were prepared using SYBR Select Master Mix (ThermoFisher, 4472903). Thermal cycling and ΔΔCt calculations were performed using an Applied Biosystems 7500 Fast Real-Time PCR System, with endogenous β-actin serving as the reference gene. Stably transfected lines expressing relatively high levels of *BMP10* were used for further analysis.

## *BMP10* expression and secretion

Stably transfected cell lines expressing *BMP10* WT and *BMP10* c.625C>T were cultured in T75 flasks (Corning) for 24 h in serum-free DMEM. The culture medium was collected, and protein extracts were prepared from the cells. Secreted BMP10 protein was isolated from culture medium using His Mag Sepharose Excel magnetic beads and His Buffer Kit (GE Healthcare, 17371220, 11003400) according to manufacturer’s instructions. Protein isolates were then concentrated for western blotting using Amicon Ultra-0.5ml 3k Centrifugal Filter devices (Millipore, UFC500308), and concentrations measured by Bradford assay (Bio-Rad, 5000201). Whole-cell lysates and concentrated media isolates were normalized using respective diluents and analyzed by western blotting. Total protein on the blots was reversibly stained using Spyro Ruby (Bio-Rad, 1703127) according to manufacturer’s instructions and visualized using the Typhoon-FLA 9500 imaging system (GE Healthcare). The blots were then probed using specific antibodies against BMP10 prodomain and growth factor domain (both 1:1000 dilution; both Thermo Scientific) and visualized by fluorescence detection using Amersham ECF Substrate (GE Healthcare) and the Typhoon imaging system. Quantitation of signal from total protein staining and BMP10-specific bands was performed using ImageQuant TL software.

## *In silico* assessment of digenic variant interaction

We ran ORVAL[19] (release 1.1), a platform that predicts the potential pathogenicity of variant combinations, on the SMYD1 p.Arg441Trp and BMP10 p.Arg209Cys variants. It assigned a classification score of 0.650 out of 1.0 and a support score of 85.40 out of 100, which placed the variant combination in the modeled 95% candidate pathogenic zone. ORVAL also incorporates results from the Digenic Effect (DE) predictor[19], which classifies pairs of variants by their probability of acting as two low pathogenicity variants (“True Digenic”), one low and one moderate pathogenicity variant (“Monogenic + Modifier”), or two strong pathogenicity variants (“Dual Molecular Diagnosis”). The DE predictor calculated a probability of 0.538 that the variant combination gives rise to a True Digenic effect, over a Monogenic + Modifier effect (p = 0.265), or a Dual Molecular Diagnosis (p = 0.197). The main feature that these tools use to model variant interaction is the biological or interactome distance between the corresponding genes, SMYD1 and BMP10. The Human Gene Connectome (HGC)[19, 20] calculates a biological distance of 10.417, which ranks at 9.4% and 16.3% of the closest direct or indirect interactions for the SMYD1 and BMP10 genes, respectively. While HGC estimates that three degrees of interaction separate SMYD1 and BMP10, String v11.0 clusters the genes within only two degrees of interaction[21].

Other coinciding variant pairs that were of interest were also assessed with ORVAL: *ROCK1* (p.Lys695*) and *MCTP2* (p.Asn22Thr) from Family 154 and *CASZ1* (p.Arg25Cys) and *ELN* (p.Gly773Asp) from Family 16. This either resulted in a weaker prediction for a True Digenic versus a Dual Molecular Diagnosis effect (0.336 versus 0.443 for the Family 154 variant pair), or no prediction at all (due to the model missing a datapoint for the gene *ELN*). These gene pairs also had larger biological distances in the HGC (16.8 for ROCK1-MCTP2, and 12.7 for ELN-CASZ1). As detailed in the main body of the manuscript, *CASZ1* became a more prominent candidate over *ELN* after further analysis.

# Supporting Note: Case Reports

**Family 16.** The proband underwent an echocardiogram at 1 day of age due to a murmur and dusky coloring. Echocardiogram showed a variant of hypoplastic left heart syndrome (HLHS) with moderate hypoplasia of the mitral valve, left ventricle, and aortic valve, a slightly constricted patent ductus arteriosus, trivial tricuspid regurgitation, and a patent foramen ovale. He underwent a hybrid approach to Stage 1 palliation with left and right pulmonary artery banding and PDA stenting at age 1 month. He later had restrictive atrial communication for which he underwent balloon atrial septostomy. At 6 months of age, he underwent a comprehensive stage II procedure (modified bidirectional Glen, Damus-Kaye-Stancel, aortic arch reconstruction, pulmonary artery repair, atrial septectomy, and removal of patent ductus arteriosus stent and pulmonary artery bands). Fontan completion was performed at 2.5 years of age. His family history is significant for a mother with bicuspid aortic valve (BAV) diagnosed at age 35 after evaluation due to the family history of left sided defects. The maternal great-grandfather (through unaffected maternal grandmother) had a BAV that required replacement at age 67.

**Family 19.** The proband is a 24-year-old male who was diagnosed with BAV with trivial AVS at age 3 years; no intervention has been required. His family history is significant for a brother diagnosed with BAV at age 3 that has not required intervention and a brother diagnosed with COA and BAV who underwent patch repair at age 4, transcatheter stent therapy for re-coarctation at age 26, then aortic valve replacement at age 27.

**Family 154.** The proband (517) was 4-year-old male at time of enrollment. He had been diagnosed with BAV and minimal AVS at age 2 that has not required intervention. His family history is significant for an identical twin brother (514) also with a history of BAV and minimal AVS diagnosed at age 2 without intervention. His mother (515) has a history of BAV with AVS and underwent valvuloplasty at age 8. The maternal uncle has a possible BAV. ES was performed on these three individuals, as well as the proband’s half-sister (518), with a mild congenital pulmonary valve anomaly without stenosis.

**Family 207.** The proband is a 9-year-old male who was diagnosed with BAV with mild dilation of the ascending aorta (2.87cm, z-score=3.48) that has not required intervention. His family history is significant for a brother who died at age 22 with BAV, accessory tissue of the mitral valve, and dilated cardiomyopathy with abnormal areas of thinned myocardium in the left ventricle (LVIDd=6.62cm, z-score=3.85, LVEF=45%), and trabeculations consistent with noncompaction. The mother was found to have mild aortic stenosis, although imaging was unable to determine if the valve was tricuspid.

**Family 238.** The proband is a 7-year-old female who was diagnosed with a small anterior muscular VSD and BAV with mild aortic root dilation (1.53cm, z-score=2.27) that has not required intervention. Her family history is significant for a sister with multiple VSDs (one apical that measured 3-4 mm and three additional each measuring 1-2 mm) as well as BAV. A maternal great aunt (through maternal grandmother) was reported to have BAV and mitral valve prolapse. The mother was reported to have a normal echocardiogram and the maternal grandmother did not have an echocardiogram.

**Family 346.** The proband was found to have a murmur on clinical exam at age 7 days. An echocardiogram showed moderate aortic stenosis and BAV with slight supravalvular narrowing. He underwent balloon valvuloplasty at age 2 weeks. His family history is significant for a sister with BAV with mild aortic stenosis that has not required intervention. The father had a normal echocardiogram by report. In the extended family history, a maternal great great uncle (through maternal grandmother and great grandmother) died in his twenties with BAV discovered on autopsy.

**Family 368.** The proband is a 2-year-old female who had an abnormal prenatal echocardiogram showing a small aorta, small mitral valve, and VSD. Immediately after birth, an echocardiogram showed coarctation of the aorta, moderate patent ductus arteriosus, BAV, moderate perimembranous VSD partially closed with aneurysmal tissue, and a small/moderate muscular VSD. She underwent surgical coarctation repair at age 2 days, a subsequent balloon dilation for residual coarctation at age 2 months, then surgical VSD closure at age 3 months. Her family history is significant for a brother with a history of a murmur and arm/leg blood pressure gradient on physical exam and coarctation of the aorta and patent ductus arteriosus on echocardiogram diagnosed at age 4 years. He underwent an aortic coarctation stent placement and patent ductus arteriosus coil occlusion at age 4 years 9 months. The family history showed a paternal great grandfather with a BAV that required a valve replacement at an unknown age. Neither parent has had an echo.

**Family 439.** The proband is a 15-year-old male who was evaluated due to family history and found to have a murmur on physical exam and a BAV found on echocardiogram. His family history is significant for a father with coarctation of the aorta and BAV diagnosed at age 13 years who underwent coarctation repair at age 18 years, and subsequently underwent an aortic valve replacement and root replacement due to ascending aortic dilation at age 39 years.

**Family 469.** The proband was a fetus delivered at 22 weeks gestation after an ultrasound at 20.5 weeks gestation showed a small left ventricle and aorta and an echocardiogram the same day showed hypoplastic left heart syndrome with mitral and aortic atresia. Karyotype showed 46,XX. The family history is significant for a father with mild aortic stenosis and BAV. Additionally, a sister was found to have severe aortic stenosis at age 2 days that required balloon dilatation at age 3 days.

**Family 528.** The proband is a 7-month-old male who presented at age 3 weeks with failure to thrive and poor ventricular function with critical coarctation of the aorta, BAV, and patent ductus arteriosus on echocardiogram. He underwent coarctation repair with division and ligation of patent ductus arteriosus at age 3 weeks. His family history is significant for a father with a history of BAV (partial fusion) diagnosed at age 26.

1. Gould RA, Aziz H, Woods CE, Seman-Senderos MA, Sparks E, Preuss C, et al. ROBO4 variants predispose individuals to bicuspid aortic valve and thoracic aortic aneurysm. Nat Genet. 2019;51(1):42-50. Epub 2018/11/21. doi: 10.1038/s41588-018-0265-y. PubMed PMID: 30455415; PubMed Central PMCID: PMCPMC6309588.

2. Stankiewicz TR, Gray JJ, Winter AN, Linseman DA. C-terminal binding proteins: central players in development and disease. Biomol Concepts. 2014;5(6):489-511. Epub 2014/11/28. doi: 10.1515/bmc-2014-0027. PubMed PMID: 25429601.

3. Hildebrand JD, Soriano P. Overlapping and unique roles for C-terminal binding protein 1 (CtBP1) and CtBP2 during mouse development. Mol Cell Biol. 2002;22(15):5296-307. Epub 2002/07/09. doi: 10.1128/mcb.22.15.5296-5307.2002. PubMed PMID: 12101226; PubMed Central PMCID: PMCPMC133942.

4. Glessner JT, Bick AG, Ito K, Homsy J, Rodriguez-Murillo L, Fromer M, et al. Increased frequency of de novo copy number variants in congenital heart disease by integrative analysis of single nucleotide polymorphism array and exome sequence data. Circ Res. 2014;115(10):884-96. Epub 2014/09/11. doi: 10.1161/circresaha.115.304458. PubMed PMID: 25205790; PubMed Central PMCID: PMCPMC4209190.

5. Scigliano S, Grégoire MJ, Schmitt M, Jonveaux PH, LeHeup B. Terminal deletion of the long arm of chromosome 10. Clin Genet. 2004;65(4):294-8. Epub 2004/03/18. doi: 10.1111/j.1399-0004.2004.00218.x. PubMed PMID: 15025722.

6. Jin SC, Homsy J, Zaidi S, Lu Q, Morton S, DePalma SR, et al. Contribution of rare inherited and de novo variants in 2,871 congenital heart disease probands. Nat Genet. 2017;49(11):1593-601. Epub 2017/10/11. doi: 10.1038/ng.3970. PubMed PMID: 28991257; PubMed Central PMCID: PMCPMC5675000.

7. Ching YH, Ghosh TK, Cross SJ, Packham EA, Honeyman L, Loughna S, et al. Mutation in myosin heavy chain 6 causes atrial septal defect. Nat Genet. 2005;37(4):423-8. Epub 2005/03/01. doi: 10.1038/ng1526. PubMed PMID: 15735645.

8. Granados-Riveron JT, Ghosh TK, Pope M, Bu'Lock F, Thornborough C, Eason J, et al. Alpha-cardiac myosin heavy chain (MYH6) mutations affecting myofibril formation are associated with congenital heart defects. Hum Mol Genet. 2010;19(20):4007-16. Epub 2010/07/27. doi: 10.1093/hmg/ddq315. PubMed PMID: 20656787.

9. Banerjee A, Vest KE, Pavlath GK, Corbett AH. Nuclear poly(A) binding protein 1 (PABPN1) and Matrin3 interact in muscle cells and regulate RNA processing. Nucleic Acids Res. 2017;45(18):10706-25. Epub 2017/10/05. doi: 10.1093/nar/gkx786. PubMed PMID: 28977530; PubMed Central PMCID: PMCPMC5737383.

10. Cha HJ, Uyan O, Kai Y, Liu T, Zhu Q, Tothova Z, et al. Inner nuclear protein Matrin-3 coordinates cell differentiation by stabilizing chromatin architecture. Nature communications. 2021;12(1):6241. Epub 2021/10/31. doi: 10.1038/s41467-021-26574-4. PubMed PMID: 34716321; PubMed Central PMCID: PMCPMC8556400.

11. Johnson JO, Pioro EP, Boehringer A, Chia R, Feit H, Renton AE, et al. Mutations in the Matrin 3 gene cause familial amyotrophic lateral sclerosis. Nat Neurosci. 2014;17(5):664-6. Epub 2014/04/02. doi: 10.1038/nn.3688. PubMed PMID: 24686783; PubMed Central PMCID: PMCPMC4000579.

12. Senderek J, Garvey SM, Krieger M, Guergueltcheva V, Urtizberea A, Roos A, et al. Autosomal-dominant distal myopathy associated with a recurrent missense mutation in the gene encoding the nuclear matrix protein, matrin 3. Am J Hum Genet. 2009;84(4):511-8. Epub 2009/04/07. doi: 10.1016/j.ajhg.2009.03.006. PubMed PMID: 19344878; PubMed Central PMCID: PMCPMC2667977.

13. Quintero-Rivera F, Xi QJ, Keppler-Noreuil KM, Lee JH, Higgins AW, Anchan RM, et al. MATR3 disruption in human and mouse associated with bicuspid aortic valve, aortic coarctation and patent ductus arteriosus. Hum Mol Genet. 2015;24(8):2375-89. Epub 2015/01/13. doi: 10.1093/hmg/ddv004. PubMed PMID: 25574029; PubMed Central PMCID: PMCPMC4380077.

14. Garg V, Muth AN, Ransom JF, Schluterman MK, Barnes R, King IN, et al. Mutations in NOTCH1 cause aortic valve disease. Nature. 2005;437(7056):270-4. doi: 10.1038/nature03940. PubMed PMID: 16025100.

15. Kerstjens-Frederikse WS, van de Laar IM, Vos YJ, Verhagen JM, Berger RM, Lichtenbelt KD, et al. Cardiovascular malformations caused by NOTCH1 mutations do not keep left: data on 428 probands with left-sided CHD and their families. Genet Med. 2016;18(9):914-23. Epub 2016/01/29. doi: 10.1038/gim.2015.193. PubMed PMID: 26820064.

16. McBride KL, Riley MF, Zender GA, Fitzgerald-Butt SM, Towbin JA, Belmont JW, et al. NOTCH1 mutations in individuals with left ventricular outflow tract malformations reduce ligand-induced signaling. Hum Mol Genet. 2008;17(18):2886-93. Epub 2008/07/03. doi: 10.1093/hmg/ddn187. PubMed PMID: 18593716; PubMed Central PMCID: PMCPMC2722892.

17. Liu Z, Yang X, Tan F, Cullion K, Thiele CJ. Molecular cloning and characterization of human Castor, a novel human gene upregulated during cell differentiation. Biochem Biophys Res Commun. 2006;344(3):834-44. Epub 2006/04/25. doi: 10.1016/j.bbrc.2006.03.207. PubMed PMID: 16631614.

18. Warner LR, Babbitt CC, Primus AE, Severson TF, Haygood R, Wray GA. Functional consequences of genetic variation in primates on tyrosine hydroxylase (TH) expression in vitro. Brain Res. 2009;1288:1-8. Epub 2009/07/14. doi: 10.1016/j.brainres.2009.06.086. PubMed PMID: 19591812.

19. Renaux A, Papadimitriou S, Versbraegen N, Nachtegael C, Boutry S, Nowe A, et al. ORVAL: a novel platform for the prediction and exploration of disease-causing oligogenic variant combinations. Nucleic Acids Res. 2019;47(W1):W93-W8. Epub 2019/05/31. doi: 10.1093/nar/gkz437. PubMed PMID: 31147699; PubMed Central PMCID: PMCPMC6602484.

20. Itan Y, Mazel M, Mazel B, Abhyankar A, Nitschke P, Quintana-Murci L, et al. HGCS: an online tool for prioritizing disease-causing gene variants by biological distance. BMC Genomics. 2014;15:256. Epub 2014/04/04. doi: 10.1186/1471-2164-15-256. PubMed PMID: 24694260; PubMed Central PMCID: PMCPMC4051124.

21. van Dam TJP, Kennedy J, van der Lee R, de Vrieze E, Wunderlich KA, Rix S, et al. CiliaCarta: An integrated and validated compendium of ciliary genes. PloS one. 2019;14(5):e0216705. Epub 2019/05/17. doi: 10.1371/journal.pone.0216705. PubMed PMID: 31095607; PubMed Central PMCID: PMCPMC6522010.
